# SCS 4th Annual Conference: Strength and Conditioning for Human Performance, Porto, Portugal, 2021

**DOI:** 10.3390/sports10060093

**Published:** 2022-06-10

**Authors:** Pedro E. Alcaraz, Tomás T. Freitas, Elena Marín-Cascales, Anthony J. Blazevich, José Oliveira, Susana Soares, João P. Vilas-Boas

**Affiliations:** 1 UCAM Research Center for High Performance Sport, Catholic University of Murcia, UCAM, 30107 Murcia, Spain; tfreitas@ucam.edu; 2 Strength and Conditioning Society, 00118 Rome, Italy; elenamcascales@gmail.com (E.M.-C.); a.blazevich@ecu.edu.au (A.J.B.); 3 Faculty of Sports, Catholic University of Murcia, UCAM, 30107 Murcia, Spain; 4 NAR—Nucleus of High Performance in Sport, São Paulo 04753060, Brazil; 5 Centre for Exercise and Sports Science Research, School of Medical and Health Sciences, Edith Cowan University, Joondalup 6027, Australia; 6 Research Centre in Physical Activity, Health and Leisure, Faculty of Sport, University of Porto, 4099-002 Porto, Portugal; joliveira@fade.up.pt; 7 Laboratory for Integrative and Translational Research in Population Health (ITR), University of Porto, 4099-002 Porto, Portugal; 8 Centre of Research, Education, Innovation and Intervention in Sport, Faculty of Sport, University of Porto, 4200-450 Porto, Portugal; susana@fade.up.pt (S.S.); jpvb@fade.up.pt (J.P.V.-B.); 9 Porto Biomechanics Laboratory, University of Porto, 4200-450 Porto, Portugal

**Keywords:** congress, performance, exercise, health, training

## Abstract

On behalf of the Strength & Conditioning Society (SCS) and the Faculty of Sport of the University of Porto, we are pleased to present the abstracts of the SCS 4th Annual Conference: Strength and Conditioning for Human Performance, which took place in, Porto, Portugal, on 12–13 November 2021. The event was a success with invited sessions from renowned international and national speakers on a myriad of topics related to strength and conditioning and its application to health and sports performance, such as agility training and testing, high-intensity interval training in chronic conditions, hamstring strain injuries in soccer, and the utilization of surface electromyography (EMG) decomposition for assessing human performance, among others. During the Conference there were also different practical workshops on (1) velocity-based training; (2) performance testing and athlete monitoring using force platforms; (3) 3D kinematics tracking and flow force assessment in aquatic sports; (4) the application of inertial sensors for physical performance testing; (5) muscle fiber recruitment, force production, and energy expenditure in progressive bicycle testing; (6) EMG decomposition, motor-units recruitment, and muscle contraction modes; and (7) recovery strategies in team-sport athletes. Researchers and academics were able to present their latest findings by submitting the abstracts that compose this Conference Report.

## 1. Introduction

The 4th Annual Conference was the first Strength & Conditioning Society (SCS) on-site meeting in over two years because of the COVID-19 pandemic. It was organized in collaboration with the Faculty of Sport of the University of Porto and took place in Porto, Portugal, on 12–13 November 2021. The event brought together the excellence of each field of research and study and provided every opportunity for delegates to learn from and contribute to the latest developments in strength and conditioning science in a stimulating social and professional setting. The Conference was planned with the risk of cancellation looming throughout due to the health and safety policies determined by the local authorities, and most decisions were made with a considerable degree of uncertainty. Still, the event was a great success with invited sessions from renowned international and national speakers on different topics related to strength and conditioning and its application to health and sports performance, such as agility training and testing, high-intensity interval training in chronic conditions, hamstring strain injuries in soccer, and the utilization of surface electromyography (EMG) decomposition for assessing human performance, among others. Moreover, there were also several practical workshops on (1) velocity-based training; (2) performance testing and athlete monitoring using force platforms; (3) 3D kinematics tracking and flow force assessment in aquatic sports; (4) the application of inertial sensors for physical performance testing; (5) muscle fiber recruitment, force production, and energy expenditure in progressive bicycle testing; (6) EMG decomposition, motor-units recruitment, and muscle contraction modes; and (7) recovery strategies in team-sport athletes. An additional point worth highlighting was the fact that researchers and academics from a multitude of countries were able to present their latest findings by submitting the abstracts that compose this Conference Report and are presented below. Finally, during the event, the SCS held a ceremony in which professional and academic excellence in the field of strength and conditioning were recognized. Namely, the Strength and Conditioning Coach of the Year Award, the Emerging Strength and Conditioning Coach of the Year Award, and the Strength and Conditioning Career Achievement Award were presented to distinguished practitioners, and the Young Investigator Award and the Applied Science Award were given to outstanding researchers.

## 2. Conference Abstracts

### 2.1. Ten-Year Longitudinal Changes in the Knee-Extension Torque–Velocity Relationship and in Maximum Muscle Power in Young, Middle-Aged, and Older Adults

**Julian Alcazar ^1,2^, Carlos Rodriguez-Lopez ^1,2^, Christophe Delecluse ^3^, Martine Thomis ^3^ and Evelien Van Roie ^3^**

^1^ GENUD Toledo Reasarch Group, Universidad de Castilla-La Mancha, Toledo, Spain; Julian.Alzar@uclm.es; Carlos.RLopez@uclm.es

^2^ CIBER of Frailty and Healthy Aging (CIBERFES), Madrid, Spain

^3^ Physical Activity, Sports and Health Research Group, Department of Movement Sciences, KU Leuven, Leuven, Belgium; Christophe.Delecluse@kuleuven.be; Martine.Thomis@kuleuven.be; Evelien.vanroie@kuleuven.be

Maximum muscle power (Pmax) is an important biomarker of physical performance in different populations ranging from athletes to older people [1]. This investigation assessed the longitudinal changes reported in young, middle-aged, and older adults in the knee extension torque–velocity (T-V) relationship and in Pmax. A total of 489 adults (311 men and 178 women, aged 19–68 years) participated in this longitudinal investigation (follow-up (median [interquartile range]) = 9.6 [9.3–10.4] years). Three age groups were created: Young (<40 years), middle-aged (40 to 59.9 years), and older (≥60 years) adults. The knee extension T-V relationship was assessed using an isokinetic dynamometer (Biodex, Shirley, New York, NY, USA) after several isometric, isotonic, and isokinetic trials. T-V data from each participant were fitted by a hybrid equation including a linear (slow to moderate velocities) and hyperbolic (moderate to fast velocities) region (R^2^ = 0.99 ± 0.02) [2]. T0 (estimated maximum isometric torque), V0 (estimated maximum unloaded velocity), and Pmax (apex of the power–velocity curve) were calculated from this equation. Linear mixed-effect models were used to assess longitudinal changes and differences between subgroups. The results showed that T0 decreased significantly over time (mean [95%CI] = −1.37 [−1.85 to −0.88] Nm per year; *p* < 0.001). There was no main effect of sex (*p* = 0.268), but there was a main effect of age group (*p* = 0.027; the decrement was significantly higher in middle-aged vs. young adults). Changes in V0 over time did not reach statistical significance (mean [95%CI] = −0.05 [−0.091 to 0.001] rad·s^−1^ per year; *p* = 0.057), and no effect of sex was detected (*p* = 0.452), while a significant main effect of age group was observed (*p* = 0.022; the decrement was significantly higher in middle-aged vs. young adults). Pmax was found to decrease significantly over time (mean [95%CI] = −5.26 [−6.52 to −4.00] W per year; *p* < 0.001), while significant main effects of sex (*p* = 0.013; the decrement was significantly higher in men vs. women) and age group (*p* = 0.004; the decrement was significantly higher in middle-aged and older vs. young adults) were also noted. To conclude, Pmax decreased in young women and men (both −0.6%·year^−1^), middle-aged women (−1.1%·year^−1^) and men (−1.4%·year^−1^), and older women (−2.4%·year^−1^) and men (−2.2%·year^−1^). The early declines in muscle power seemed to be caused primarily by mechanisms related to force production, while the progressive decline in muscle power was associated with both force- and velocity-related mechanisms at a later age. These findings might be of high relevance to prescribe resistance training recommendations to the general population.

**Funding:** This study was conducted under the authority of the First and Third waves of the Flemish Policy Research Center on Sport and was funded by the Flemish Government. E. Van Roie was supported by the Research Foundation Flanders, Belgium (senior postdoctoral fellowship 12Z5720N). J. Alcazar was supported by the Biomedical Research Networking Center on Frailty and Healthy Aging (CIBERFES) and FEDER funds from the European Union (CB16/10/00477).

**References**

1. Foldvari, M.; Clark, M.; Laviolette, L.C.; Bernstein, M.A.; Kaliton, D.; Castaneda, C.; Pu, C.T.; Hausdorff, J.M.; Fielding, R.A.; Singh, M.A. Association of muscle power with functional status in community-dwelling elderly women. *J. Gerontol. A Biol. Sci. Med. Sci.* **2000**, *55*, M192–M199. https://doi.org/10.1093/gerona/55.4.m192.
2. Rodriguez-Lopez, C.; Beckwee, D.; Luyten, F.P.; Van Assche, D.; Van Roie, E. Reduced knee extensor torque production at low to moderate velocities in postmenopausal women with knee osteoarthritis. *Scand. J. Med. Sci. Sports* **2021**, *31*, 2144–2155. https://doi.org/10.1111/sms.14035.

### 2.2. Effects of a Multicomponent Exercise Training Program on Lean Mass Loss Attenuation after Bariatric Surgery

**Giorjines Boppre ^1,2^, Florêncio Diniz-Sousa ^1,2^, Lucas Veras ^1,2^, Vitor Devezas ^4^, John Preto ^4^, Hugo Santos-Sousa ^4^, Leandro Machado ^2,3^, João Paulo Villas-Boas ^2,3^, José Oliveira ^1,2^ and Hélder Fonseca ^1,2^**

^1^ Research Centre in Physical Activity, Health and Leisure (CIAFEL), Faculty of Sport, University of Porto, Porto, Portugal; giorjines_boppre@hotmail.com

^2^ Laboratory for Integrative and Translational Research in Population Health (ITR), Porto, Portugal

^3^ Porto Biomechanics Laboratory (LABIOMEP)

^4^ Centro de Responsabilidade Integrado de Obesidade (CRIO), Centro Hospitalar Universitário de São João, Porto

Bariatric surgery is the most common surgical treatment performed worldwide for patients with severe obesity, resulting in substantial and sustained weight losses [1]. Nevertheless, reductions in adiposity resulting from bariatric surgery are accompanied by other detrimental consequences on body composition, namely substantial lean mass losses that can have both functional and metabolic negative consequences [2,3]. Our aim was to determine whether a multicomponent exercise training program (MExT) attenuates post-BS lean mass loss. This is an ancillary study of a randomized clinical trial registered at ClinicalTrials.gov (NCT02843048). Sixty-six patients (43.0 ± 9.8 years) who underwent BS (body mass index 44.6 ± 5.5 kg/m^2^) were allocated to either a control (CG; bariatric surgery + standard medical care; n = 21) or an exercise group (EG; bariatric surgery + standard medical care + MExT; n = 45) and were followed for 1 year. MExT started 1 month after BS and consisted of 3 d/week, 75-min-duration multicomponent exercise sessions that included multidirectional jumps, balance, and resistance exercises. Body weight, whole-body lean mass, appendicular lean mass, and lower limb lean mass were assessed by dual-energy X-ray absorptiometry (Hologic Explorer QDR) at pre-surgery, 1, 6, and 12 months after BS. The effect of exercise on the selected body composition outcomes was tested by linear mixed models. Data were reported as the estimated mean and standard error. Compared to baseline, both the CG and EG, at the end of the first year after bariatric surgery, showed a significant reduction in body weight (CG: −41.1 ± 1.8 kg, *p* < 0.001; EG: −40.0.5 ± 1.3 kg, *p* < 0.001), whole-body lean mass (CG: −10.20 ± 0.7 kg, *p* < 0.001; EG: −8.52 ± 0.5 kg, *p* < 0.001), appendicular lean mass (CG: −4.84 ± 0.4 kg, *p* < 0.001; EG: −4.02 ± 0.3 kg, *p* < 0.001), and lower limb lean mass (CG: −3.76 ± 0.3 kg, *p* < 0.001; EG: −3.24 ± 0.2 kg, *p* < 0.001). At the end of the first year after BS, in comparison with the CG, participation in a MExT induced whole-body lean mass loss attenuation (CG = 43.4 kg vs. EG = 44.8 kg; ∆ = −1.4 kg, *p* = 0.036). No differences were observed between groups at 12 months after surgery for the remaining body composition outcomes assessed. One year after bariatric surgery, significant reductions in all of the analyzed body composition outcomes were observed. Nevertheless, participation in MExT after bariatric surgery reduced whole-body lean mass losses, therefore being an effective strategy to attenuate lean mass losses after bariatric surgery.

**Funding:** Giorjines Boppre, Florêncio Diniz-Sousa and Lucas Veras are supported by the FCT grants SFRH/BD/146976/2019, SFRH/BD/117622/2016 and UI/BD/150673/2020, respectively. The Research Centre in Physical Activity, Health, and Leisure (CIAFEL) is funded by Regional Development Fund (ERDF) through COMPETE and by FCT grant (FCT/UIDB/00617/2020). Recent research by our group on this subject has been supported by FCT grant PTDC/DTP-DES/0968/2014.

**References**

1. Jabbour, G.; Salman, A. Bariatric Surgery in Adults with Obesity: The Impact on Performance, Metabolism, and Health Indices. *Obes. Surg.* **2021**, *31*, 1767–1789.
2. O’Brien, P.E.; Hindle, A.; Brennan, L.; Skinner, S.; Burton, P.; Smith, A.; Crosthwaite, G.; Brown, W. Long-Term Outcomes After Bariatric Surgery: A Systematic Review and Meta-analysis of Weight Loss at 10 or More Years for All Bariatric Procedures and a Single-Centre Review of 20-Year Outcomes After Adjustable Gastric Banding. *Obes. Surg.* **2019**, *29*, 3–14.
3. Boppre, G.; Diniz-Sousa, F.; Veras, L.; Oliveira, J.; Fonseca, H. Can exercise promote additional benefits on body composition in patients with obesity after bariatric surgery? A systematic review and meta-analysis of randomized controlled trials. *Obes. Sci. Pract.* **2022**, *8*, 112–123.

### 2.3. Acute Effect of Tennis Point Duration in Shoulder Torque Production and Muscular Balance

**André Brito, Diogo Carvalho, Pedro Fonseca, Sofia Monteiro, Aléxia Fernandes and Ricardo Fernandes**

Centre of Research, Education, Innovation and Intervention in Sport and Porto Biomechanics Laboratory, Faculty of Sport, University of Porto, 4200-450 Porto, Portugal

Tennis forehand success depends on the combination of accuracy and ball speed [1]. In longer games, the influence from peripheral fatigue is higher, reducing stroke functionality [2] and changing the movement kinematics [3]. Since tennis systematic actions affect different joints, leading to unilateral musculoskeletal imbalances and injuries [4], we aimed to analyze the acute effect of a tennis point in shoulder rotation torque production and muscular balance. Six male and four female competitive tennis players (20.5 ± 1.6 vs. 14.5 ± 0.5 years, 68.1 ± 6.9 vs. 49.2 ± 4.9 kg of body weight and 176.9 ± 7.7 vs. 160.9 ± 4.6 cm of height, respectively) voluntarily performed 10 external/internal shoulder rotation repetitions of both upper limbs at 180°/s on an isokinetic dynamometer, before and after five forehand tennis points using a ball launcher defined at 72 km·h^−1^. A paired-samples *t*-test was used for the upper limbs comparisons before (M1) and after (M2) the played point considering strength-related variables (*p* < 0.05). The dominant upper limb presented higher torque, power, and work than the non-dominant at M1 and M2 for internal rotation (absolute peak torque: M1 38.7 ± 17.0 vs. 31.3 ± 11.9, M2 36.4 ± 15.9 vs. 30.5 ± 10.8 N·m; relative peak torque: M1 61.9 ± 16.8 vs. 51.1 ± 13.6, M2 58.2 ± 15.3 vs. 50.1 ± 12.1 N·m; average peak torque: M1 35.5 ± 16.7 vs. 27.5 ± 10.7, M2 33.6 ± 15.3 vs. 26.7 ± 9.8 N·m; total work: M1 612.3 ± 298.0 vs. 405.1 ± 179.0, M2 588.7 ± 263.3 vs. 419.3 ± 176.3 J; average power: M1 72.5 ± 39.4 vs. 47.6 ± 23.3, M2 68.4 ± 33.5 vs. 48.3 ± 22.6 W, respectively) and external rotation (absolute peak torque: M1 27.4 ± 10.1 vs. 20.6 ± 6.7, M2 27.4 ± 8.9 vs. 20.2 ± 7.0 N·m; relative peak torque: M1 44.8 ± 10.4 vs. 34.2 ± 7.2, M2 44.8 ± 7.1 vs. 33.3 ± 7.2 N·m; average peak torque: M1 23.3 ± 9.6 vs. 17.4 ± 5.2, M2 23.4 ± 8.6 vs. 17.7 ± 6.1 N·m; total work: M1 281.6 ± 84.2 vs. 216.2 ± 84.2, M2 259.4 ± 97.8 vs. 215.3 ± 86.9 J; average power: M1 32.8 ± 15.9 vs. 25.4 ± 11.6, M2 29.6 ± 13.2 vs. 24.6 ± 11.7 W, respectively). The internal/external ratios were higher in the dominant upper limb only at M2 (79.6 ± 12.2 vs. 68.4 ± 16.1%). Furthermore, the indexes of symmetry showed that only one or two tennis players (depending on the variable) presented indexes < 10% (rating from 0.3 to 58.6%). The five forehands led to a decrease in the dominant shoulder internal rotation absolute, relative, and average peak torque and tend to increase the internal/external ratio (74.7 ± 15.0 vs. 79.6 ± 12.2%) but showed no influences on the internal or external rotation of the non-dominant upper limb. Due to the extreme use of the dominant upper limb during practice, tennis players show substantially contralateral asymmetry in shoulder rotation, with this being important to implementing compensatory strength training ensuring muscular balance and joint health. The dominant limb torque and power production decrease after five forehands demonstrates the influence of the number of shots per point in strength levels, possibly decreasing forehand effectiveness.

**Funding:** This research received no external funding.

**References**

1. Kolman, N.; Huijgen, B.; Kramer, T.; Elferink-Gemser, M.; Visscher, C. The dutch technical-tactical tennis test (D4T) for talent identification and development: Psychometric characteristics. *J. Hum. Kinet*. **2017**, *55*, 127–138.
2. Wang, L.-H.; Lo, K.-C.; Su, F.-C. Skill level and forearm muscle fatigue effects on ball speed in tennis serve. *Sports Biomech.* **2019**, *4*, 419–430.
3. Kwon, S.; Pfister, R.; Hager, R.; Hunter, I.; Seeley, M. Influence of tennis racquet kinematics on ball topspin angular velocity and accuracy during the forehand groundstroke. *J. Sports Sci. Med.* **2017**, *16*, 505–513.
4. Ellenbecker, T.; Roetert, E.P. Age specific isokinetic glenohumeral internal and external rotation strength in elite junior tennis players. *J. Sports Sci. Med.* **2003**, *6*, 63–70.

### 2.4. Is Running Kinematics Affected by Wearing a Lower Jaw Repositioning Splint?

Filipa Cardoso ^1,2,^*, João P. Lima ^1,2^, Ricardo Cardoso ^1,2^, João Paulo Vilas-Boas ^1,2^, João C. Pinho ^3,4^, David B. Pyne ^5^ and Ricardo J. Fernandes ^1,2^

^1^ Centre of Research, Education, Innovation and Intervention in Sport, CIFI2D, Faculty of Sport, University of Porto, Portugal; anafg.cardoso@hotmail.com; up201701745@edu.fade.up.pt; davidrcardoso@gmail.com; jpvb@fade.up.pt; ricfer@fade.up.pt

^2^ Porto Biomechanics Laboratory, LABIOMEP-UP, Faculty of Sport, University of Porto, Portugal; anafg.cardoso@hotmail.com; up201701745@edu.fade.up.pt; davidrcardoso@gmail.com; jpvb@fade.up.pt; ricfer@fade.up.pt

^3^ Faculty of Dental Medicine, University of Porto, Portugal; pinhojc53@gmail.com

^4^ Institute of Science and Innovation in Mechanical and Industrial Engineering, INEGI, Faculty of Engineering, University of Porto, Portugal; pinhojc53@gmail.com

^5^ Research Institute for Sport & Exercise, University of Canberra, Canberra, Australia; David.Pyne@canberra.edu.au

Acute ergogenic effects of using jaw repositioning splints during exercise have been reported both on aerobic [1] and anaerobic [2] variables. However, far less attention has been spent on analyzing the effect of these devices on body kinematics [3]. We quantified the effect of repositioning the lower jaw on running kinematics in the low to severe exercise intensity domains. Ten high-level middle-distance male runners (age 26.8 ± 5.7 years, height 180 ± 6 cm and body mass 68 ± 9 kg) were recruited to perform (48 h apart) a 7 × 800 m incremental running protocol twice on a 400 m outdoor track field, with 1 km·h^−1^ increments and 30 s rest periods. The velocity was controlled by audible feedback signals every 100 m. To evaluate the effect of changing the lower jaw position, a placebo (without jaw repositioning) and a lower jaw repositioning splint were custom-made and manufactured for each participant and tested under counterbalanced conditions. Kinematic data were collected at 120 Hz using two high-definition video cameras (GoPro HERO6 Black, San Mateo, CA, USA) placed on a 100 m track field section. For each running intensity, six consecutive strides were analyzed (Kinovea, version 0.8.27, Boston, MA, USA) and averaged to assess the stride time, frequency, and length for each participant. Strides were also divided to obtain the step time of each foot. A paired *t*-test was performed to compare the kinematic variables obtained between the two different experimental conditions, and between feet in each experimental condition (with the significance level set at *p* ≤ 0.05). The values of stride time, frequency, and length were similar between the placebo and jaw repositioning conditions across all the studied running intensities. However, when wearing the repositioning splint, the stride length presented a tendency to be lower in the low-intensity domain (2.93 ± 0.33 vs. 2.97 ± 0.34 m, *p* = 0.08, Cohen’s d = 0.6). Despite no differences in stride time between experimental conditions, the step time performed by each foot in the placebo exhibited 2–3% asymmetry (e.g., 0.33 ± 0.01 vs. 0.32 ± 0.02 s) across all the evaluated intensities. When wearing the repositioning splint, the step time asymmetry between feet was only observed at the heavy-intensity domain (~3%). Although changing the lower jaw position did not improve stride-related variables, no negative effects were observed, and wearing a repositioning splint led to more symmetric step times than placebo. The ergogenic effects on aerobic and anaerobic performance of wearing repositioning splints reported in the literature [1,2], concurrently with our findings on the step time symmetry and the lack of detrimental effects on running kinematics, might encourage runners to trial this type of oral device. However, further studies should be conducted from a biophysical perspective to better understand the effects of changing the lower jaw position during exercise.

**Funding:** This research was funded by the Fundação para a Ciência e a Tecnologia (FCT), grant number 2020.05012.BD.

**References**

1. Schultz Martins, R.; Girouard, P.; Elliott, E.; Mekary, S. Physiological responses of a jaw-repositioning custom-made mouthguard on airway and their effects on athletic performance. *J. Strength Cond. Res.* **2020**, *34*, 422–429. https://doi.org/10.1519/JSC.0000000000002679.
2. Cesanelli, L.; Cesaretti, G.; Ylaitė, B.; Iovane, A.; Bianco, A.; Messina, G. Occlusal Splints and Exercise Performance: A Systematic Review of Current Evidence. *Int. J. Environ. Res. Public Health* **2021**, *18*, 10338. https://doi.org/10.3390/ijerph181910338.
3. Maurer, C.; Stief, F.; Jonas, A.; Kovac, A.; Groneberg, D.A.; Meurer, A.; Ohlendorf, D. Influence of the Lower Jaw Position on the Running Pattern. *PLoS ONE* **2015**, *10*, e0135712. https://doi.org/10.1371/journal.pone.0135712.

### 2.5. Multicomponent Online Exercise Training Improved Lower Body Strength of Older Adults Who Used to Exercise in In-Person Exercise Programs Prior to the Outbreak

Susana Carrapatoso ^1,2^, Pedro Abdalla ^1,3^, Carla Cadete ^4,5^, Joana Carvalho ^1^, Maria Paula Santos ^1^ and Lucimére Bohn ^1,2,^*

^1^ Research Center in Physical Activity, Health and Leisure (CIAFEL), Faculty of Sports, University of Porto (FADEUP), Portugal Laboratory for Integrative and Translational Research in Population Health (ITR), Porto, Portugal; scarrapatoso@fade.up.pt

^2^ Faculty of Psychology, Education and Sport, University Lusófona of Porto, Porto, Portugal

^3^ College of Nursing of the University of Sao Paulo at Ribeirao Preto (EERP/USP), Brazil

^4^ Research Center Hei-Lab—The Playful Interaction Design Lab, Porto, Portugal

^5^ School of Communication, Architecture, Arts and Information Technologies, University Lusófona of Porto, Porto, Portugal

The current aim is to evaluate the effectiveness of an online-exercise program on physical fitness in community-dwelling older adults. This is a non-randomized controlled trial study comprising 62 older adults (74.59 ± 5.79 yrs, 59.7% women) who used to exercise in the in-person exercise program Mais Ativos, Mais Vividos, hosted in the Faculty of Sports, University of Porto. Because the in person-program was interrupted as a consequence of the outbreak, older adults were invited to integrate into an intervention group (IG). Those participants who did not could follow the online program constituted the control group (CG). The IG group was submitted to an 8-month online exercise program (3 sessions/week, multicomponent regime). Physical fitness (senior fitness test) was assessed prior to and after the intervention. Changes over time were modeled using a linear mixed model. The IG did not improve physical fitness. After adjustments for age, sex, and body fat, there was a minimal significant benefit for lower body strength favoring the IG (group × time interaction 1.55 (0.65); *p* = 0.020). The online exercise program did not improve overall physical fitness, but a slight improvement was observed concerning lower body strength.

**Funding:** This work was supported by the Portuguese Foundation for Science and Technology: CIAFEL—Research Center in Physical Activity, Health, and Leisure (grant no. FCT/UIDB/00617/2020).

### 2.6. Kinematic Analysis of the Basketball Jump Shot Comparing Two Shooting Distances

André Caseiro ^1,^*, Cíntia França ^1,2^, Ana Faro ^1^ and Beatriz B. Gomes ^1,3^

^1^ University of Coimbra, Faculty of Sport Sciences and Physical Education, Coimbra, Portugal

^2^ LARSYS, Interactive Technologies Institute, 9020 105 Funchal, Portugal

^3^ University of Coimbra, Research Unit for Sport and Physical Activity CIDAF (uid dtp/042143/2020), Coimbra, Portugal

* Correspondence: afgcaseiro@gmail.com

The basketball jump shot is the shooting technique players use the most in a game of basketball. The outcome of the jump shot is mostly determined by the height, velocity, and angle of ball release [1]. Considering that the shooting distance has an impact on the kinematic variables of height, velocity, and the angle of ball release, players need to adjust their technique depending on the shooting distance to the basket [2,3]. We try to identify how increases in shooting distance reflect upon these kinematic variables and what strategies players use to overcome the increasing shooting distance. Nine male federated athletes participated. Each player performed three successful jump shots from the Free-throw (FT) line and three successful jump shots from the Three-point (3P) line. In each player, eight markers were placed to allow the digitalization of the body segments. The data were obtained through video recording (120 Hz) of the sagittal plane of movement, and exported using Tracker software, which allowed for calculation of the kinematic variables. We also calculated the position of the center of mass (CoM), which allows for the calculation of the height of the CoM at the moment of ball release, the peak height of CoM during the jump shot, and the horizontal displacement of the CoM. With the increase in shooting distance, we observed a decrease in the height of ball release, from 2.45 ± 0.09 m for the FT line to 2.37 ± 0.08 m in the 3P line. The angle of ball release also decreases with the increase in shooting distance, with 53.00 ± 5.47° at the FT line compared to 51.50 ± 4.65° at the 3P line. The velocity of ball release shows an increase with the increase in shooting distance, with 6.76 ± 0.43 m·s^−1^ for the jump shots performed at the FT line compared to 8.16 ± 0.42 m·s^−1^ for the jump shots performed at the 3P line. The results lead us to understand that an increase in shooting distance shows a decrease in the release height and angle of ball release and shows an increase in the velocity of ball release. The increase in the release velocity is necessary to overcome the larger shooting distance [3]. With the increase in shooting distance, we also observed an increase in the horizontal displacement of the CoM towards the basket, which shows that players try to reduce the shooting distance when jumping [4]. With the increase in shooting distance we observed a decrease in the height of ball release, although the peak height of the CoM increased with the increase in shooting distance, from 1.33 ± 0.07 m reached in jump shots from the FT line to 1.37 ± 0.07 from the 3P line. This may suggest that the release of the ball occurs at an earlier moment in jump shots performed further from the basket; therefore, it is suggested that players takes advantage of the impulsion generated in the jump to transfer energy to the ball.

**Funding:** This research received no external funding.

**References**

1. Miller, S.; Bartlett, R. The relationship between basketball shooting kinematics, distance and playing position. *J. Sports Sci.* **1996**, *14*, 243–253, https://doi.org/10.1080/02640419608727708.
2. Button, C.; Macleod, M.; Sanders, R.; Coleman, S. Examining movement variability in the basketball free-throw action at different skill levels. *Res. Q. Exerc. Sport* **2003**, *74*, 257–269, https://doi.org/10.1080/02701367.2003.10609090.
3. Okazaki, V.H.A.; Rodacki, A.L.F. Increased distance of shooting on basketball jump shot. *J. Sport. Sci. Med.* **2012**, *11*, 231–237. https://doi.org/https://www.ncbi.nlm.nih.gov/pubmed/24149195.
4. Podmenik, N.; Supej, M.; Kugovnik, O.; Erculj, F. Movement of the body’s Centre of Mass During a Jump Shot Related To the Distance From the Basket of Young Players. *Kinesiol. Slov.* **2015**, *21*, 21–33.

### 2.7. The Effects of Repeated Sprints with and without Rapid Horizontal Decelerations on Residual Neuromuscular Fatigue in Professional Male Footballers

**Daniel D Cohen ^1,2^, Juliano Spineti ^3,4^, Antônio P F Neto ^3^, Gabriel Vianna ^3^, Daniel F De Souza ^5^, Rob Gathercole ^6^, Damian J Harper ^7^ and Matt Taberner ^8,9^**

^1^ Masira Research Institute, Faculty of Health Sciences, University of Santander, Bucaramanga, Colombia; danielcohen1971@gmail.com

^2^ Sports Science Centre (CCD), Colombian Ministry of Sport (Mindeporte), Colombia

^3^ Fluminense Football Club, Rio de Janeiro, Brazil; Juliano.spineti@hotmail.com; pedro.farias.neto@hotmail.com; gabriel-cm@hotmail.com

^4^ University of Coimbra, Faculty of Sport Sciences and Physical Education, Research Unit in Sport and Physical Activity (CIDAF)

^5^ VALD Performance, Albion, Australia; d.souza@vald.com

^6^ lululemon, Futures Innovation, Vancouver, Canada; rjgathercole@gmail.com

^7^ Institute of Coaching and Performance, School of Sport and Health Sciences, University of Central Lancashire, UK; d.harper5@uclan.ac.uk 

^8^ Performance and Medical Department, Orlando Magic Basketball Club, Orlando, USA; matthewtaberner@btinternet.com

^9^ School of Sport and Exercise Sciences, Liverpool John Moores University, Liverpool, UK

High-intensity decelerations are an important component of football match-play [1]. Their exceptional mechanical demands may contribute substantially to muscle damage and associated residual neuromuscular fatigue observed 24 to 48 h post-match but may also promote adaptations that enhance performance and/or the capacity to recover from repeated decelerations [2]. We aimed to determine the specific impact of repeated rapid horizontal decelerations on residual fatigue by comparing changes in countermovement jump (CMJ) performance following repeated sprint efforts that did or did not include rapid decelerations. Sixteen male professional U23 footballers were randomly assigned to an on-field repeated sprint protocol (2 bouts of 10 × 50 m; mean peak speed 28.3 km·h (7.86 m·s^−1^)), with each 50 m sprint followed by either a rapid deceleration (stop within 3 m) (RSDEC, n = 8) or a slower deceleration within 15 m (RS, n = 8). We compared performances in CMJs performed just prior to and 48 h post the RS protocols using a *t*-test and the effect size (ES). At 48 h post, the RSDEC group displayed significant (*p* < 0.01) moderate-to-large-magnitude changes in the concentric duration (ES = 1.17), eccentric deceleration duration (ES = 0.85), eccentric deceleration rate of force development (ES = −0.72), concentric impulse at 100 ms. And concentric rate of power development at 100 ms (ES = −0.79, −1.05, respectively), while there were no significant changes in the jump height or concentric and eccentric deceleration impulses. In the RS group, there were no significant changes in any variables. These results suggest that repeated sprint efforts that end with rapid deceleration result in significant residual neuromuscular fatigue, indicated by alterations in specific time-constrained eccentric and concentric CMJ kinetic variables. Performing the same number of sprint efforts with a minimal deceleration demand did not lead to alterations in any of the CMJ variables assessed. Our findings reinforce the importance of management of the deceleration component of player running loads in training, and recovery from both competitive matches and training with a large deceleration load. Our analysis also demonstrates the value of a comprehensive analysis of CMJ kinetics in the detection of residual fatigue induced by repeated decelerations.

**Funding:** This research received no external funding.

**References**

1. Harper, D.J.; Carling, C.; Kiely, J. High-Intensity Acceleration and Deceleration Demands in Elite Team Sports Competitive Match Play: A Systematic Review and Meta-Analysis of Observational Studies. *Sports Med*. **2019**, *49*, 1923–1947. https://doi.org/10.1007/s40279-019-01170-1.
2. Harper, D.J.; Kiely, J. Damaging nature of decelerations: Do we adequately prepare players? *BMJ Open Sport Exerc. Med.* **2018**, *4*, e000379. https://doi.org/10.1136/bmjsem-2018-000379.

### 2.8. PAPE Responses Are Delayed When a Complex-Training Set Is Added to the Warm-Up

**Francisco Cuenca-Fernández ^1,2^, Óscar López-Belmonte ^1^, Jesús Juan Ruiz-Navarro ^1^, Ana Gay ^1^, Esther Morales-Ortíz ^1^, Gracia López-Contreras ^1^ and Raúl Arellano ^1^**

^1^ Aquatics Lab, Departmet of Physical Education and Sports, Faculty of Sport Sciences, Ctra Alfacar SN (18011), Granada, University of Granada; cuenca@ugr.es

^2^ Strength and Conditioning Society, Rome, Italy

To maximize performance, it has been suggested that a general warm-up should include an aerobic dynamic component performed at an intensity of ˂60% of the individual’s VO2max, as well as a skill-specific component or conditioning activities, in which the athlete practices the specific movement to be performed [1]. However, there appears to be a current tendency in the literature to assume that any improvement in performance following conditioning activities is due to post-activation performance enhancement (PAPE) and does not take into account the possible prior benefits of the warm-up activities [2,3]. The aim of this study was to determine if the warm-up that is usually performed in competition could be improved after including a set of complex training activies. Nine elite swimmers (men = 5 and women = 4) were tested in two warm-up conditions on two different days. One of them consisted of a swimming standard warm-up of 2000 m composed of varied swimming exercises (SWU), drills, and all-out trials intercalated with three swimming starts. The other one consisted of the same warm-up as SWU followed by a complex-training set (CTWU (five maximal eccentric squat repetitions on an adapted versa-pulley, followed by ten split-squat jumps)). Within the three swimming starts performed during the warm-up, the horizontal velocity of the hip during flight (VxH) was averaged and considered the baseline. In the five and fifteen minutes after the end of the in-water warm-up, swimmers were asked to perform two maximal swimming starts. A two-way repeated-measures ANOVA (intervention [SWU, CTWU] × time [Pre, Post_5min, Post_10min]) was used to explore the timeline performance differences between the protocols. Pair comparisons were verified with th Bonferroni post-hoc test. Statistical significance was set at *p* < 0.05. There was a significant time interaction (F = 3.828; *p* = 0.044) and intervention × time interaction (F = 10.750; *p* = 0.001). In SWU, the VxH in Pre and Post_5min was similar, but there was a decrease in performance in Post_10min (Pre: 4.76 ± 0.23 m/s; Post_5min: 4.77 ± 0.24 m/s; Post_10min: 4.62 ± 0.24 m/s). In CTWU, there was a decrease in VxH at Post_5min compared to Pre; however, there was a return to baseline in Post_10min (Pre: 4.75 ± 0.22 m/s; Post_5min: 4.59 ± 0.29 m/s; Post_10min: 4.77 ± 0.20 m/s). In terms of superior performance, none of the warm-ups proved to produce better results in the swim start than the other. However, such results were better in either case depending on the time at which the task was registered. As fatigue and PAPE coexist, the intensity/volume of the conditioning activity could play an important role in preparing athletes for competition, as more intense activities could delay the time course of performance improvements and shift the window of opportunity to perform the sport task later. This could be particularly relevant in swimming, as competitors have to wait in the call room (≤20 min) between their warm-up and the race.

**Funding:** This research was funded by the Spanish Research Agency and European Regional Development Funds (ERDF), grant number PGC2018-102116-B-I00 “SWIM II: Specific Water Innovative Measurements: applied to the improvement in performance”.

**References**

1. Bishop, D. Warm-up II: Performance changes following active warm-up and how to structure the warm-up. *Sports Med.* **2003**, *33*, 483–498. https://doi.org/10.2165/00007256-200333070-00002.
2. Cuenca-Fernández, F.; Smith, I.C.; Jordan, M.J.; MacIntosh, B.R.; López-Contreras, G.; Arellano, R.; Herzog, W. Nonlocalized postactivation performance enhancement (PAPE) effects in trained athletes: A pilot study. *Appl. Physiol. Nut. Met.* **2017**, *42*, 1122–1125. https://doi.org/10.1139/apnm-2017-0217.
3. Young, W.B.; Behm, D.G. Effects of running, static stretching and practice jumps on explosive force production and jumping performance. *J. Sports Med. Phys. Fit.* **2003**, *43*, 21–27. PMID: 12629458.

### 2.9. Evolution in the Body Composition of a Professional Football Player after a Nutritional Intervention

**Alejandro Del Águila-Sánchez ^1^, Sergio Porcel-Gámez ^1^, Javier Conde-Pipo ^2^, Alba Blas-Díaz ^2^, Alejandro López-Moro ^2^, Leticia Cantero-Ballesteros ^2^ and Miguel Mariscal-Arcas ^2^**

^1^ High Performance Sports Research Centre of Alex del Aguila, Roquetas de Mar, Almeria, Spain

^2^ Research Group of Mmhealthscience, Dpt. Nutrition and Food Science, School of Pharmacy, University of Granada, Spain

Sports nutrition is a relatively new area of study whose objective is the application of nutritional and food science principles to the improvement of sports performance. Correct nutrition is an essential factor to maintaining the health of a professional football player, but there are also various dietary factors that can influence biomechanical, psychological, and physiological factors [1,2,3,4]. For example, the loss of excess body fat may improve biomechanical efficiency on the field [5,6]. It is rather common for coaches and athletes to focus on nutrition only during certain training or competition periods, without realizing that for optimal sporting performance it is essential to eat and nourish oneself correctly at all times [7]. The aim of this study was to intervene for one year with a continuous ideal Mediterranean Diet (MD) model (balance, equilibrium, variety) without modifying the training pattern of a professional football player in the Spanish football league, observing the changes caused in the body composition of the athlete. The intervention and data collection were carried out during 2020 and 2021. The adjustment to the diet was daily, balancing their energy intake in relation to the daily training load, with a nutritional balance within the FAO/WHO recommendations and including all food groups as dictated by the diet quality indices [8]. Different diet quality indices were also used for the overall quantitative assessment: DQI-I, adapted by Mariscal-Arcas et al. (2007), and NQI, specific to athletes and proposed by our research group in 2015 [9,10]. The initial weight of the subject was 71.10 kg, with a height of 184 cm, BMI = 21.00 kg/m^2^, and %fat = 10.34%. The highest values reached after the nutritional intervention were 75.70 kg weight, BMI = 22.36 kg/m^2^, and %fat = 9.46%. All the values indicate an improvement in the body composition of this professional footballer after the intervention for one year with an ideal Mediterranean Diet (MD) model, which could be the most appropriate way to optimize sports performance from a nutritional health point of view.

**Funding:** This study was supported by the Andalusian Regional Government (Nutrition, Diet and Risk Assessment AGR255), FEDER-ISCIII PI14/01040 and Consejería de Transformación Económica, Industria, Conocimiento y Universidades, Junta de Andalucía P18-RT-4247.

**References**

1. Scaramella, J.; Kirihennedige, N.; Broad, E. Key nutritional strategies to optimize performance in Para athletes. *Phys. Med. Rehabil. Clin. N. Am.* **2018**, *29*, 283–298.
2. Nutrition and athletic performance. *Med. Sci. Sports Exerc.* **2016**, *48*, 543–568.
3. Mountjoy, M.; Sundgot-Borgen, J.; Burke, L.; Ackerman, K.E.; Blauwet, C.; Constantini, N.; Lebrun, C.; Lundy, B.; Melin, A.; Meyer, N.; et al. International Olympic committee (IOC) consensus statement on relative energy deficiency in sport (RED-S): 2018 update. *Int. J. Sport. Nutr. Exerc. Metab.* **2018**, *28*, 316–331.
4. Pramuková, B.; Szabadosová, V.; Soltésová, A. Current knowledge about sports nutrition. *Australas. Med. J.* **2011**, *4*, 107–110.
5. Gutiérrez, R.; Aldea, L.; Cavia, M.D.M.; Alonso-Torre, S.R. Relation between the body composition and the sports practice in teenagers. *Nutr. Hosp.* **2015**, *32*, 336–345.
6. Guisado, J.P. Rendimiento deportivo: Composición corporal, peso, energía-macronutrientes y digestión (II). *Arch. Med. Deporte* **2009**, *16*, 451–459.
7. Jenner, S.L.; Buckley, G.L.; Belski, R.; Devlin, B.L.; Forsyth, A.K. Dietary intakes of professional and semi-professional team sport athletes do not meet sport nutrition recommendations-A systematic literature review. *Nutrients* **2019**, *11*, 1160.
8. De Expertos OMS/FAO I de UCM. DIETA, NUTRICIÓN Y PREVENCIÓN DE ENFERMEDADES CRÓNICAS [Internet]. Who.int. Available online: http://apps.who.int/iris/bitstream/handle/10665/42755/WHO_TRS_916_spa.pdf;jsessionid=F759DC62C4F20C2A4627F2F4ADCE82E6?sequence=1 (accessed on 11 November 2021).
9. Mariscal-Arcas, M.; Romaguera, D.; Rivas, A.; Feriche, B.; Pons, A.; Tur, J.A.; Olea-Serrano, F. Diet quality of young people in southern Spain evaluated by a Mediterranean adaptation of the Diet Quality Index-International (DQI-I). *Br. J. Nutr.* **2007**, *98*, 1267–1273.
10. Palacin-Arce, A.; Monteagudo, C.; de Dios Beas-Jimenez, J.; Olea-Serrano, F.; Mariscal-Arcas, M. Proposal of a nutritional quality index (NQI) to evaluate the nutritional supplementation of sportspeople. *PLoS ONE* **2015**, *10*, e0125630.

### 2.10. Countermovement Jump Kinetics and Prospective Risk of Anterior Knee Pain in Male Professional Volleyball Players

**Alejandro del Águila Sánchez ^1^, Jarrod L Antflick ^2^, Jose Luis Hernández Davó ^3^ and Daniel D. Cohen ^4,5^**

^1^ Research Department, Alex del Águila Personal Training Center, Spain; a.delaguila.sanchez@gmail.com

^2^ Imperial College, London, UK; jarrod@total-performance.com

^3^ Faculty of Health Sciences, University Isabel I, Spain; jlhdez43@gmail.com

^4^ Faculty of Health Sciences, University of Santander, Colombia; danielcohen1971@gmail.com

^5^ Sports Science Centre (CCD), Colombian Ministry of Sport (Mindeporte), Colombia

Anterior knee pain (AKP) is a common complaint in sports involving repeated jumps such as volleyball. Within the diagnosis of AKP, 45% of volleyball players are diagnosed with patella tendinopathy (PT) [1]. While a number of modifiable risk factors have been identified for AKP, associations between CMJ kinetics and AKP has not been evaluated. We aimed to determine whether CMJ kinetics are associated with the risk of knee pain during the competitive season in elite volleyball based on a retrospective analysis of 71 male professional players. CMJ kinetics from a start-of-season assessment (five hands-on-hips trials) were compared in 35 players who reported knee pain (AKP) with 36 who did not (NKP). There were significant (*p* < 0.001) large to very large effect size (ES) differences between AKP and NKP in the eccentric deceleration phase duration (AKP: 182 ± 19; NKP: 148 ± 19; ES = 1.79), flight time:contraction time (AKP: 0.73 ± 0.1; NKP: 0.84 ± 0.1; ES = 1.20), eccentric deceleration rate of force development (AKP: 84.3 ± 19.6; NKP: 124.6 ± 25.7; ES = 1.23), concentric peak force (AKP: 25.3 ± 2.2; NKP: 28.0 ± 2.3; ES = 1.21), concentric rate of power development@100 ms (AKP: 118.2 ± 40.6; NKP: 185.9 ± 69.9; ES = 1.18), concentric duration (AKP: 303 ± 30; NKP: 268 ± 32; ES = 1.13), and concentric impulse@100 ms (AKP: 115.2 ± 15.2; NKP: 129.0 ± 17.6; ES = 0.83). There were no significant between-group differences in jump height, concentric peak power, and concentric or eccentric deceleration impulse. Eccentric and concentric variables associated with efficient stretch shortening cycle function and rapid force production/reduction, as represented by shorter-phase durations and higher rates of force/power development, appeared to be protective against experiencing knee pain during the season. In contrast, jump height, concentric peak power, and overall phase impulses were not associated with risk. While none of the players reported knee pain at the time of the CMJ assessment, the differences observed could potentially be the result of existing mechanical changes, which subsequently manifested as knee pain during the season. Further work with a more robust diagnostic criteria of AKP and tendinopathy is needed to confirm the interpretation of these findings.

**Funding:** This research received no external funding.

**Reference**

1. Lian, Ø.; Engebretsen, L.; Bahr, R. Prevalence of Jumper’s Knee among Elite Athletes from Different Sports: A Cross-sectional Study. *Am. J. Sports Med.* **2005**, *33*, 561–567.

### 2.11. Relationship between Service Velocity and ASH Test Performance in Professional Volleyball Players

**Alejandro del Águila Sánchez ^1^, Ben Ashworth ^2^ and Daniel D. Cohen ^3^**

^1^ Research Department, Alex del Águila Personal Training Center, Spain; a.delaguila.sanchez@gmail.com

^2^ Sports Science & Medicine Department, AC Sparta Praha, Czech Republic; bennyashworth@gmail.com

^3^ Faculty of Health Sciences, University of Santander, Colombia; danielcohen1971@gmail.com

The athletic shoulder (ASH) test is a series of maximal isometric shoulder strength assessments performed at three different shoulder extension positions (I, Y, T) [1]. These assessments are a means to profile and monitor underlying neuromuscular performance in athletes/sports with an overhead/shoulder force production component that is critical to competition outcomes, such as the serve in volleyball [2]. We aimed to determine whether ASH test I position (180° shoulder abduction) force–time variables were associated with service velocity in elite volleyball players. Nineteen (n = 19) professional players (mean weight 88.3 ± 8.3 kg) performed three dominant-arm trials of the I position ASH test on a force plate with 30 s of rest between trials. Two days later, following a specific volleyball service warm-up, the players performed five maximal volleyball serves with ball velocity measured using a radar gun. The mean serve velocity was 103 ± 7.46 (km·h). Spearman’s correlation was used to examine potential relationships between service velocity (maximal performance in each) and ASH test force–time variables: Absolute (ABS) and bodyweight-adjusted (BW) peak force (PF), and the rate of force development (RFD) epochs of 0–50, 0–100, 0–150, and 0–200 ms. Correlations between velocity and force–time variables were RFD0-50: ABS; r = 0.62 *p* = 0.005, BW; r = 0.57 *p* = 0.010, RFD0-100: ABS r = 0.60, *p* = 0.007; BW r = 0.61 *p* = 0.006, RFD0-150: ABS r = 0.39 *p* = 0.095; BW r = 0.34 *p* = 0.150, RFD0-200: ABS r = 0.27, *p* = 0.26; BW r = 0.20 *p* = 0.42 and peak force: ABS r = −0.18 *p* = 0.46; ABS r = −0.21, *p* = 0.38. Specific ASH-I position variables representative of the early rate of force production (RFD0-50, RFD0-100) significantly correlated with the service velocity (Spearman’s r > 0.6 and *p* values < 0.05), but peak force and RFD0-200 did not. These findings demonstrate the importance of, or association between, early rapid isometric force production characteristics and an important dynamic high velocity activity within volleyball and relevance to athlete profiling in the sport. This simple-to-perform isometric test may be a useful monitoring tool for volleyball players’ upper body “readiness” for, or neuromuscular response to, competitive matches. Future research should examine potential associations between improvements or decrements in I position variables and changes in service velocity.

**Funding:** This research received no external funding.

**References**

1. Ashworth, B.; Hogben, P.; Singh, N.; Tulloch, L.; Cohen, D.D. The Athletic Shoulder (ASH) test: Reliability of a novel upper body isometric strength test in elite rugby players. *BMJ Open Sport Exerc. Med.* **2018**, *4*, e000365.
2. Forthomme, B.; Croisier, J.L.; Ciccarone, G.; Crielaard, J.M.; Cloes, M. Factors correlated with vol-leyball spike velocity. *Am. J. Sports Med.* **2005**, *33*, 1513–1519.

### 2.12. Analysis of Physical Demand in the Brazilian Soccer Championship in the 2020/21 Season Using Main Components

**Rodrigo dos Santos Guimarães ^1^, Vitor Bertoli Nascimento ^2^, Tomas García-Calvo ^1^, David Lobo-Triviño ^1^, Ana Rubio-Morales ^1^ and Juan José Pulido ^1^**

^1^ Facultad de Ciencias del Deporte, Universidad de Extremadura; rodrigo.sportcoach@gmail.com; tgarciac@unex.es; davidlobo123@gmail.com; anrubiom@unex.es; jjpulido@unex.es

^2^ Department of Physical Education, Universidade de Londrina; vitorbertolinascimento@yahoo.com.br

With the use of new technology in sport, a great amount of information is generated in each training session and competition. Among these technologies, the use of GPS is widespread in soccer. For a better understanding of the complexity and interrelation of the physical demands of games and training, the use of machine learning techniques as main components is recommended. This technique allows for a reduction in the number of variables, information about the contribution of each of the variables created to explain the phenomenon, in addition to the contribution of the original variables in the reduced variables, allowing compression of the dynamics of physical demand. The aim of the present study was to describe the physical demands of professional soccer players at different stages of competition through the analysis of principal components (PCA). PCA was used to reduce ten variables: Rating of perceived exertion (RPE), meters covered per minute, total distance covered, distance covered at high intensity, the distance between 4 m/s and 5.5 m/s, number of sprints > 23 km/h, number of accelerations, and number of decelerations, and training impulse (TRIMP). The sample consisted of 35 soccer players from a professional Brazilian soccer team that played in the second division of the national league, with 38 games analyzed. Two main components were created, and these account for 71.6% and 15.7% for the first round and 72.7% and 14.7% for the second shift. The variables that most contributed to the first component in the first round were deceleration (14.27%), total distance (14.14%), acceleration (13.51%), and distance covered at 4 m/s to 5.5 m/s (13.46%), and for the second component, they were meters per minute (60.44%) and RPE (12.81%). For the second round, a greater contribution was observed for the distance covered between 4 m/s and 5.5 m/s, with a reduction in the contribution of the number of accelerations for the first component, deceleration (14.22%), total distance covered (13.97%), distance covered from 4 m/s to 5.5 m/s (14.33%), and acceleration (12.23%), and for the second component, meters per minute and RPE in addition to the reduction of the contribution of RPE (10.95%) for the second component. Such changes in the contributions of physical demand variables in the main components suggest a modification in the team’s tactical behavior, which caused a reduction in the acceleration contribution and an increase in the numbers of the variable 14.4 to 19.8 km/h. Thus, we can conclude that the use of the technique of main components helps to understand the physical demands of soccer games, with an alteration between the first and second phases of competition being observed in the investigated sample.

**Funding:** This research was funded by the Ministry of Science and Innovation (Government of Spain, grant number IJC2019-040788-I” and “by European regional development fund and Government of Extremadura (Spain, grant number GR18102).

### 2.13. Influence of Undulating vs. Traditional Microcycle Periodization on the Weekly External Loads and Match Day’s Readiness Level in Elite Academy Soccer Players

Tom Douchet ^1,2,^*, Christos Paizis ^2^ and Nicolas Babault ^2^

^1^ Center for Performance Expertise, CAPS, U1093 INSERM, Sport Science Faculty, University of Bourgogne-Franche-Comté, 3 allée des Stades Universitaires, BP 27877, CEDEX, 21078 Dijon, France; tom.douchet@egmail.com (T.D.); christos.paizis@u-bourgogne.fr (C.P.); nicolas.babault@u-bourgogne.fr (N.B.)

^2^ Dijon Football Côte d’Or (DFCO), 17 rue du Stade, 21000 Dijon, France

* Correspondence: tom.douchet@egmail.com; Tel.: +33-3-80-39-67-01

In the context of academy soccer teams, coaches aim to develop players’ physical qualities. The literature demonstrates that the most demanding sessions (MDS), supposed to enhance players’ development, are usually performed four and three days before match day (MD-4 and MD-3) during traditional (TRAD) soccer periodization [1]. However, other sports implement undulating periodization (UND) with their most demanding sessions on MD-4 and MD-2, and a low-load session on MD-3 [2]. The low load session on MD-3 could therefore increase players’ readiness level on the second-most demanding session (MDS2) and lead to increased external loads. Performed week after week, these increased external loads could potentiate long-term physical development. However, the distance between MDS2 and MD could lead to a decreased readiness level on MD. Therefore, this study aimed to investigate the impact of UND on players’ external loads on MDS2, and their readiness level on MD in comparison to TRAD. Twenty-eight elite academy soccer players from the U17 and U19 teams were tested over 5 weeks. The contents of weeks 1 and 4 were strictly similar and were supposed to lead to comparable physical levels on the tested weeks (weeks 2 and 5). Weeks 2 and 5 were randomized with UND (MDS1 on MD-4 and MDS2 on MD-2) or TRAD (MDS1 on MD-4 and MDS2 on MD-3). TRAD and UND implemented similar contents during weeks 2 and 5. However, MD-3 and MD-2 contents were reversed on UND in comparison to TRAD. MDS2 was a strength/speed-oriented session supposed to enhance high-speed running and sprint running. Players wore a 15-Hz GPS and rated their perceived exertion (RPE) for each session. Tests were conducted on MD-4 for baseline values (PRE) and repeated before (POST) a friendly match on MD. Tests consisted of a counter-movement jump (CMJ), 20 m sprint, Illinois agility test (IAT), and Hooper questionnaire (for the subjective fitness level). Statistical significance was set at *p* < 0.05. On MDS2, players reported similar total distance, distance in 0–15 km/h and 15–20 km/h thresholds, number of accelerations >3 m/s^−2^, decelerations <−3 m/s^−2^, and RPE between TRAD and UND. However, players reported greater distances during UND than TRAD in 20–25 km/h (306.36 m ± 117.11 vs. 223.52 m ± 92.28, *p* < 0.05) and >25 km/h (89.56 m ± 44.86 vs. 67.21 m ± 44.58, *p* < 0.05). Tests revealed identical values for PRE between the two tested weeks for CMJ, 10 m, 20 m, and IAT. For POST, CMJ, 20 m, and IAT were similar between UND and TRAD. However, the 10 m (1.89 s ± 0.10 vs. 1.92 s ± 0.09, *p* < 0.05) and Hooper index (7.90 A.U. ± 2.14 vs. 9.50 A.U. ± 3.44, *p* < 0.05) tests reported lower values in UND compared to TRAD. The results of the present study demonstrated that UND can be a good alternative to TRAD as it can lead to increased distances in high-speed and sprint running. Furthermore, in comparison to TRAD, UND does not lead to increased fatigue on MD. 

**Funding:** The present study was financed by the Center for Performance Expertise.

**References**

1. Douchet, T.; Paizis, C.; Babault, N. Typical weekly physical periodization in French academy soccer teams: A survey. *unpublished*.
2. Dubois, R.; Lyons, M.; Paillard, T.; Maurelli, O.; Prioux J. Influence of weekly workload on physical, biochemical and psychological characteristics in professional rugby union players over a competitive season. *J. Strength Cond. Res.* **2020**, *34*, 527–545. https://doi.org/10.1519/JSC.0000000000002741.

### 2.14. Influence of the Order of the Physical Qualities during the Microcycle on the Weekly External Loads and Match Day’s Readiness Level in Elite Academy Soccer Players

Tom Douchet ^1,2,^*, Christos Paizis ^2^ and Nicolas Babault ^2^

^1^ Center for Performance Expertise, CAPS, U1093 INSERM, Sport Science Faculty, University of Bourgogne-Franche-Comté, 3 allée des Stades Universitaires, BP 27877, CEDEX, 21078 Dijon, France; tom.douchet@egmail.com (T.D.); christos.paizis@u-bourgogne.fr (C.P.); nicolas.babault@u-bourgogne.fr (N.B.)

^2^ Dijon Football Côte d’Or (DFCO), 17 rue du Stade, 21000 Dijon, France

* Correspondence: tom.douchet@egmail.com; Tel.: +33-3-80-39-67-01

Weekly periodization is of paramount importance to plan for short- and long-term performance. Therefore, coaches try to optimize the training content and order in which they implement them. Previous unpublished observations [1] demonstrated that undulating periodization increased the high-intensity external loads without additional performance decrements on match day (MD). Practitioners usually implement aerobic sessions during the early- mid-week, and strength/speed sessions during mid- late-week [2]. However, the order in which physical qualities are implemented during the week could be questioned. This study aimed to investigate whether the order of the physical qualities influenced the external loads and the subsequent readiness level on MD. Thirty-two elite academy soccer players from the U17 and U19 teams were tested over five weeks. The contents of weeks 1 and 4 were strictly similar and were supposed to lead to comparable physical levels on the tested weeks (weeks 2 and 5). Weeks 2 and 5 were randomized with Aerobic/Strength-Speed periodization (AER/SS) (Aerobic session (AER) on MD-4 and Strength-Speed (SS) on MD-2), or Strength-Speed/Aerobic (SS/AER) periodization (SS on MD-4 and AER on MD-2). AER/SS and SS/AER implemented similar contents during weeks 2 and 5. Players wore a 15-Hz GPS and rated their perceived exertion (RPE) for each session. Tests were conducted on MD-4 for baseline values (PRE) and repeated before (POST) a friendly match on MD. Tests consisted of a counter-movement jump (CMJ), 20 m sprint, Illinois agility test (IAT), and Hooper questionnaire (for the subjective fitness level). Statistical significance was set at *p* < 0.05. For AER, players reported similar total distance, distance in 0–15 km/h and >25 km/h thresholds, number of accelerations >3 m/s^−2^, decelerations <−3 m/s^−2^, and RPE between AER/SS and SS/AER. However, players reported greater distances during AER/SS than SS/AER in 15–20 km/h (1273.53 m ± 328.51 vs. 1174.84 m ± 210.33, *p* < 0.05) and 20–25 km/h (658.92 m ± 264.41 vs. 478.17 m ± 259.10, *p* < 0.01). For SS, players reported similar total distance, distance in 0–15 km/h and 15–20 km/h thresholds, number of accelerations >3 m/s^−2^, decelerations <−3 m/s^−2^, and RPE between AER/SS and SS/AER. However, players reported greater distances during SS/AER than AER/SS in 20–25 km/h (298.84 m ± 120.12 vs. 223.24 m ± 114.86, *p* < 0.05) and >25 km/h (110.74 m ± 34.65 m vs. 84.96 ± 43.85, *p* < 0.05). Tests revealed identical values for PRE and POST between the two tested weeks for CMJ, 20 m, and IAT. The results of the present study demonstrated that the order of the sessions does not lead to significant differences in readiness level on MD. Additionally, players were shown to be more efficient during the early week. Therefore, coaches should place the physical quality they consider most important first.

**Funding:** The present study was financed by the Center for Performance Expertise.

**References**

1. Douchet, T.; Paizis, C.; Babault, N. Influence of the Order of the Physical Qualities during the Microcycle on the Weekly External Loads and Match Day’s Readiness Level in Elite Academy Soccer Players. *unpublished*.
2. Douchet, T.; Paizis, C.; Babault, N. Typical weekly physical periodization in French academy soccer teams: A survey. *unpublished*.

### 2.15. Cyclic Velocity Fluctuation in Elite- and Good-Level Backstrokers 

**Aléxia Fernandes ^1^, Susana Soares ^1^, Francisco Silva ^1^, Bruno Mezêncio ^2^, João Paulo Vilas-Boas ^1^ and Ricardo J. Fernandes ^1^**

^1^ Centre of Research, Education, Innovation and Intervention in Sport and Porto Biomechanics Laboratory, Faculty of Sport, University of Porto, Porto, Portugal; alexiaafernandes@gmail.com; susana@fade.up.pt; frantsilva@gmail.com; jpvb@fade.up.pt; ricfer@fade.up.pt

^2^ Biomechanics Laboratory, School of Physical Education and Sport, University of São Paulo, São Paulo, Brazil; brunomezencio@msn.com

Cyclic movements are known to display a repeating structure due to their continuity, although fluctuation can be accepted, producing similar but not identical repetitive cycles [1]. Backstroke swimming is a cyclic and continuous movement, with repetitive upper and lower limbs propulsive actions. Attending to the aquatic environment particularities, velocity fluctuation may be higher [2], but these constraints are likely dealt with differently among swimmers, depending on their flexibility skills, i.e., performance level. The current study aimed to assess cyclic velocity fluctuation in elite- and good-level swimmers performing the backstroke technique. Four elite- and four good-level male backstrokers (17.0 vs. 16.3 ± 1.0 years old, 177.5 ± 1.9 vs. 179.5 ± 3.5 cm height, 65.1 ± 3.8 vs. 67.5 ± 10.8 kg weight and 709 ± 155 vs. 402 ± 50 FINA points, respectively) performed, in a 25 m indoor pool, a 25 m maximal backstroke and were recorded in the sagittal plane using underwater and aerial cameras (fixed to a setup able to be pushed alongside the pool). Velocimetric data were assessed, and calculations of biomechanical variables (mean velocity, minimum and maximum relative to mean velocities, intra-cycle velocity variation, stroke index, stroke rate, stroke length, indexes of coordination, and synchronization) were performed and compared using an independent-samples *t*-test. The intraclass correlation coefficient assessed the intra-subject velocity cycles correlation (*p* < 0.05). Elite swimmers presented higher mean velocity and lower stroke rates than the good-level swimmers (1.70 ± 0.11 vs. 1.47 ± 0.11 m·s^−1^ and 0.89 ± 0.05 vs. 0.74 ± 0.07 cycles·s^−1^), but no differences were found in minimum (0.74 ± 0.02 vs. 0.77 ± 0.02) and maximum (1.30 ± 0.03 vs. 1.27 ± 0.02) relative velocities, intra-cycle velocity variation (13.4 ± 1.48 vs. 13.7 ± 0.96), stroke index (3.28 ± 0.24 vs. 2.94 ± 0.32), stroke length (1.93 ± 0.04 vs. 1.99 ± 0.14 m·cycle), index of coordination (−7 ± 5 vs. −7 ± 9%), and synchronization (−0.01 ± 0.14 vs. 0.01 ± 0.02). Nevertheless, good-level swimmers presented smoother and more identical velocity cycles in-between, with ICC = 0.92 (0.80–0.99 95%CI) vs. 0.51 (0.18–0.83 95%CI). The similarity between performance levels regarding the majority of the studied biomechanical variables suggests a loss of information when using mean values, standard deviations, and coefficients of variation to describe motion characteristics, influencing the variability assessment by diminishing the chances of detecting existing differences. In fact, elite swimmers showed higher cyclic velocity fluctuation likely due to their system flexibility to explore different strategies to find the most effective action. Higher plasticity improves the ability to face aquatic environment constraints and, therefore, performance variability is an optimality and adaptability indicator.

**Funding:** This research was funded by Fundação para a Ciência e Tecnologia (FCT), grant number DFA/BD/6799/2020.

**References**

1. Preatoni, E.; Hamill, J.; Harrison, A.J.; Hayes, K.; Van Emmerik, R.E.; Wilson, C.; Rodano, R. Movement variability and skills monitoring in sports. *Sports Biomech.* **2013**, *12*, 69–92. https://doi.org/10.1080/14763141.2012.738700.
2. Bartlett, R.; Wheat, J.; Robins, M. Is movement variability important for sports biomechanists? *Sports Biomech.* **2007**, *6*, 224–243. https://doi.org/10.1080/14763140701322994.

### 2.16. Effects of Multicomponent Training and Detraining Period on Lower Limb Relative Power of Older People with Limited Functional Capacity

Ángel Iván Fernández-García ^1^, Alejandro Gómez-Bruton ^1^, Ana Moradell ^1^, David Navarrete-Villanueva ^1^, Marcela González-Gross ^2^, José Gerardo Vila-Vicente ^3^, Jorge Pérez-Gómez ^4^, Ignacio Ara ^5^, José Antonio Casajús ^1^, Alba Gómez-Cabello ^1^ and Germán Vicente-Rodríguez ^1,^*

^1^ GENUD (Growth, Exercise, NUtrition and Development) Research Group, Universidad de Zaragoza, Zaragoza, Spain

^2^ ImFine Research Group, Facultad de Ciencias de la Actividad Física y del Deporte-INEF, Universidad Politécnica de Madrid, Madrid, Spain

^3^ VALFIS Research Group, Instituto de Biomedicina (IBIOMED), Facultad de Ciencias de la Actividad Física y del Deporte, Universidad de León, Spain

^4^ HEME (Health, Economy, Motricity and Education) Research Group, Universidad de Extremadura, Cáceres, Spain

^5^ GENUD (Growth, Exercise, NUtrition and Development) Toledo Research Group, Universidad de Castilla-La Mancha, Toledo, Spain

* Correspondence author: gervicen@unizar.es

Muscle power is a predictor of functional limitations and frailty in older adults, and it declines at a faster rate compared to muscle mass or strength [1]. Therefore, this study aimed to analyze the effects of a 6-month multicomponent training (MCT) and the consequences of a 4-month detraining period on relative lower-limb power of older adults with limited functional capacity. A total of 65 older adults (47 women; 81.1 ± 6.0 years) were divided into a control (CON: 24) or training group (TRAIN) following a quasi-experimental design. The TRAIN performed a 6-month MCT, while CON continued with their usual lifestyle. In order to participate in the study, participants had to be older than 65 years and present a score between 4 and 9 points in the Short Physical Performance Battery. Lower limb relative power was assessed by validated equations [2], taking into account the time needed to complete the five sit-to-stand repetitions test and using participants’ height and chair height. Evaluations were performed at baseline, at the end of the MCT (6-month), and after the detraining period (10-month). Analyses of covariance (ANCOVA) for repeated measures, adjusting by gender and age, were performed to evaluate the effects of training and detraining on power. The TRAIN improved their relative lower limb power after MCT (absolute change: 1.26 ± 0.10 W·kg^−1^) and decreased it after the detraining period (absolute change: −0.56 ± 0.09 W·kg^−1^; all *p* < 0.05). Nevertheless, the level after detraining was higher than baseline (absolute change: 0.70 ± 0.10 W·kg^−1^; *p* < 0.05). On the other hand, CON showed an improvement after 6-month and 10-month periods with respect to the baseline (absolute change: 0.54 ± 0.13 W·kg^−1^ and 0.45 ± 0.14 W·kg^−1^, respectively; both *p* < 0.05). Between-groups comparisons showed that TRAIN obtained better performance than CON after MCT (3.12 ± 0.13 W·kg^−1^ vs. 2.29 ± 0.17 W·kg^−1^; *p* < 0.005), although no differences were found between groups after the detraining period. Group-by-time interactions were found for the 6-month MCT, the following 4 months of detraining, and the whole 10-month study (all *p* < 0.05). Although, to a lesser extent, the CON participants also improved their power, perhaps due to the familiarization with the test, higher motivation derived from the healthy lifestyle talks, or other unknown causes. The MCT seems to be an effective strategy to improve relative lower-limb power in older adults with functional limitations. The 4-month detraining period reduced the magnitude of the improvement witnessed immediately after the MCT, although this was still significantly superior to baseline levels. Result suggests that the shorter the non-training period, the better the maintenance of the benefits of training.

**Funding:** The authors are grateful to all collaborators: The nursing homes, health centers, and participants involved in the EXERNET-Elder 3.0 project and council social services, whose cooperation and dedication made this study possible. A. I. F. G received a PhD grant from the Spanish Government (BES-2017-081402). A.M. received a PhD grant from “Gobierno de Aragón” (2016–2021). D.N.-V. received a grant from “Gobierno de Aragón” (DGAIIU/1/20). This study was funded by “Ministerio de Economía, Industria y Competitividad” (DEP2016-78309-R), “Centro Universitario de la Defensa de Zaragoza” (UZCUD2017-BIO-01), the Biomedical Research Networking Center on Frailty and Healthy Aging (CIBERFES), and FEDER funds from the European Union (CB16/10/00477).

**References**

1. Baltasar-Fernández, I.; Alcázar, J.; Mañas, A.; Alegre, L.M.; Alfaro-Acha, A.; Roríguez-Mañanas, L.; García-García, F.J.; Losa-Reyna, J. Relative sit-to-stand power cut-off points and their association with negatives outcomes in older adults. *Sci. Rep.* **2021**, *11*, 19460.
2. Alcázar, J.; Losa-Reyna, J.; Rodríguez-López, C.; Alfaro-Acha, A.; Rodríguez-Mañas, L.; Ara, I.; García-García, F.J.; Alegre, L.M. The sit-to-stand muscle power test: An easy, inexpensive and portable procedure to assess muscle power in older people. *Exp. Gerontol.* **2018**, *112*, 38–43.

### 2.17. Does the Angle of Directional Change Affect the Relationships between Linear Sprint, Curve Sprint, and Change of Direction Performance in Elite Young Soccer Players?

**Tomás T. Freitas ^1,2,3,4,5^, Pedro E. Alcaraz ^1,5^, Santiago Zabaloy ^6,7^, Elena Marín-Cascales ^5^, Lucas A. Pereira ^3,4^ and Irineu Loturco ^3,4,8^**

^1^ UCAM Research Center for High Performance Sport, Catholic University of Murcia (UCAM), Murcia, Spain

^2^ Faculty of Sport Sciences, Catholic University of Murcia (UCAM), Murcia, Spain

^3^ Nucleus of High Performance in Sport (NAR), São Paulo, Brazil

^4^ Department of Human Movement Sciences, Federal University of Sao Paulo, São Paulo, Brazil

^5^ Strength and Conditioning Society, Rome, Italy

^6^ Faculty of Physical Activity and Sports, University of Flores, Buenos Aires, Argentina

^7^ Faculty of Sports Sciences, Universidad Pablo de Olavide, Seville, Spain

^8^ University of South Wales, Pontypridd, Wales, UK

Sprinting actions are crucial and decisive in elite soccer and are characterized by their multidirectional nature [1]. For this reason, to assess athletes, practitioners frequently utilize different multidirectional sprint tests, some requiring rapid and aggressive decelerations and directional changes (e.g., 505 test) and others focused on less sharp maneuvers (e.g., Zigzag test) and curved sprint (CS) actions [2]. The aim of this study was to investigate the relationships between linear (5, 10- and 17-m) and different multidirectional sprint speed tests (CS, Zigzag, 505) in soccer players. Thirty-nine male young elite soccer players (16.3 ± 0.7 years) from a first-division Brazilian professional team’s academy performed linear sprint, CS, Zigzag, and 505 change of direction (COD) tests. A Pearson product–moment analysis was performed to determine the correlations among the distinct variables. Moderate to large associations were observed between linear (5-, 10- and 17-m) and CS (r = 0.53–0.88; *p*< 0.05) and Zigzag performances (r = 0.36–0.40; *p*< 0.05) and between CS and Zigzag (r = 0.42; *p*< 0.05). Conversely, no meaningful correlations were found between 505 and linear sprint (r = 0.16–0.24; *p* > 0.05), CS (r= 0.15–0.20; *p* > 0.05) and Zigzag (r = 0.24; *p* > 0.05) tests. The present results indicate that shallow directional changes (i.e., Zigzag: 90°–100°) were associated with linear and CS (in which velocity maintenance is key), whereas more aggressive COD (i.e., 505: 180°) were not. The movement patterns (e.g., body lateral inward lean and thigh separation at touchdown) and force application characteristics (e.g., increased mediolateral ground reaction forces) in the Zigzag and CS may have potentially impacted, at least in part, the relationships identified. The 505, conversely, is mainly characterized by greater braking requirements and considerable ankle dorsiflexion, knee and hip flexion, and trunk lean angles [3], which may explain why significant correlations with CS and Zigzag results were not observed. In conclusion, it seems that exercises aimed at improving the ability to perform COD actions of ~90° may potentially result in an enhanced capability to sprint along curvilinear paths, which may not be the case with drills focused on sharp COD maneuvers that require higher deceleration efforts and a greater reduction of the velocity of the center of mass.

**Funding:** This research received no external funding.

**References**

1. Bloomfield, J.; Polman, R.; O’Donoghue, P. Physical demands of different positions in FA Premier League soccer. *J. Sports Sci. Med.* **2007**, *6*, 63.
2. Loturco, I.; Pereira, L.A.; Fílter, A.; Olivares-Jabalera, J.; Reis, V.P.; Fernandes, V.; Freitas, T.T.; Requena, B. Curve Sprinting Soccer: Relationship with linear sprints and vertical jump performance. *Biol. Sport* **2020**, *37*, 277.
3. Dos’ Santos, T.; McBurnie, A.; Thomas, C.; Comfort, P.; Jones, P.A. Biomechanical determinants of the modified and traditional 505 change of direction speed test. *J. Strength Cond. Res.* **2020**, *34*, 1285–1296.

### 2.18. Body Composition Is an Important Influencing Factor of Sprinting and Jumping Performance in Youth Amateur Rugby Players

**Tomás T. Freitas ^1,2,3^, Julián Giráldez ^4^, Braian Fink ^4^, Pedro E. Alcaraz ^1^, Lucas A. Pereira ^3,5^, Irineu Loturco ^3,5,6^ and Santiago Zabaloy ^4,7^**

^1^ UCAM Research Center for High Performance Sport, Catholic University of Murcia (UCAM), Murcia, Spain

^2^ Faculty of Sport Sciences, Catholic University of Murcia (UCAM), Murcia, Spain

^3^ Nucleus of High Performance in Sport (NAR), São Paulo, Brazil

^4^ Faculty of Physical Activity and Sports, University of Flores, Buenos Aires, Argentina

^5^ Department of Human Movement Sciences, Federal University of Sao Paulo, Sao Paulo, Brazil

^6^ University of South Wales, Pontypridd, Wales, UK

^7^ Faculty of Sports Sciences, Pablo de Olavide University, Seville, Spain

Rugby Union is a physically demanding game, in which well-developed physical qualities (e.g., body size, strength, speed, and aerobic fitness) are important attributes for player success [1,2,3]. However, to date, there is a limited understanding of how these qualities differ between playing positions and age categories (i.e., Under-17 (U17) and Under-19 (U19)) in amateur players, and whether meaningful associations exist between the aforementioned variables. Thus, this study aimed to compare the body composition and physical qualities of U17 and U19 forwards (FW) and backs (B), and examine the correlations between body composition, jump, sprint, and relative strength performances. Fifty-two young male rugby players (U17 (n = 30): 16.2 ± 0.6 years and U19 (n = 22): 18.09 ± 0.9 years) performed the following assessments: Anthropometrics (height and body mass (BM)) and body composition (∑3 skinfolds (SF), body fat (BF), and lean body mass (LM)), vertical jump, 30-m sprint, and squat one-repetition maximum (1RM SQ). The differences between positions and categories (U17 FW vs. U17 B vs. U19 FW vs. U19 B) were analyzed through a one-way ANOVA, with a post-hoc Bonferroni analysis used to determine the origin of any differences. A Pearson product–moment analysis was performed to determine the correlations between the distinct variables. Significant differences were observed for BM and body composition variables among playing positions independently of the age category (*p*: 0.05 to < 0.001; ES: 0.72 to 2.24). Regarding physical qualities, U17 FW showed significantly different performances across all variables when compared to U19 B (*p*: 0.05 to < 0.001; ES: 1.08 to 3.21). Moreover, U17 FW showed lower relative strength (*p*: 0.05–0.095; ES: 1.66 to 1.88) and slower 30-m sprint times (*p*: 0.024–0.018; ES: 0.57 to 1.82) when compared to U17 B and U19 FW. Overall, in B, significant correlations were observed between BM and LM and all the other variables (r: −0.72 to 0.50). Conversely, for FW, BF and SF were mostly related to vertical jump and sprint times (r: −0.62 to 0.52) but not to relative strength. In conclusion, clear differences exist between playing positions (i.e., B vs. FW) independently of the age category, but no differences were observed when comparing U17 B and U19 B. Concerning body composition, LM may represent an important indicator for strength, jump, and sprint performances among B. Lastly, for FW, SF and BF were related to jump and sprint performance. The present findings may have important implications for talent identification and long-term player development. Practitioners are recommended to monitor these body composition variables due to the level of association found with explosive actions (i.e., sprinting and jumping).

**Funding:** This research received no external funding.

**References**

1. Darrall-Jones, J.D.; Jones, B.; Till, K. Anthropometric and physical profiles of English academy rugby union players. *J. Strength Cond. Res.* **2015**, *29*, 2086–2096.
2. Jones, B.; Weaving, D.; Tee, J.; Darrall-Jones, J.; Weakley, J.; Phibbs, P.; Read, D.; Roe, G.; Hendricks, S.; Till, K. Bigger, stronger, faster, fitter: The differences in physical qualities of school and academy rugby union players. *J. Sports Sci.* **2018**, *36*, 2399–2404.
3. Duthie, G.M. A framework for the physical development of elite rugby union players. *Int. J. Sports Physiol. Perform.* **2006**, *1*, 2–13.

### 2.19. Which Physical Performance Test Is Best Associated with an Improved Vitality in Employees of a Spanish Multinational Company?

Paola Gómez-Redondo ^1,^*, Javier Leal-Martín ^1,2^, Victoria Marín ^3^, Pilar Urdiola ^3^, Ignacio Ara ^1,2^ and Asier Mañas ^1,2^

^1^ GENUD Toledo Research Group, University of Castilla-La Mancha, Toledo, Spain; *paola.gomez1@alu.uclm.es

^2^ CIBER of Frailty and Healthy Aging (CIBERFES), Madrid, Spain

^3^ Media and People Services Department. Health Area, Grupo Red Eléctrica de España SAU, Madrid, Spain

The aim of the present work was to analyze which physical performance test is best associated with the level of vitality in workers. In this cross-sectional study, 287 company workers (100 women; 45.5 ± 10.1 years; BMI: 25.5 ± 4.2 kg/m^2^) participated. Vitality was measured using the Short Form-36 Health Survey (SF-36). In addition, the following physical performance tests were assessed: (1) Handgrip test, (2) CMJ jump, (3) Mcardle step test, (4) sit-and-reach, and (5) Flemish balance. A Spearman correlation analysis was performed for the physical performance tests and vitality. Tests that correlated significantly (*p* < 0.05) were subsequently entered into a linear regression model, which was adjusted for various covariates (gender, age, BMI and number of diseases). The two physical performance tests that correlated significantly with vitality level were the handgrip test (ICC = 0.245; *p* ≤ 0.001) and the CMJ jump test (ICC = 0.119; *p* = 0.044); however, no significant correlation was found for time on the step (ICC = 0.074; *p* = 0.213), sit-and-reach (ICC = −0.037; *p* = 0.537), and Flemish balance (ICC = 0.033; *p* = 0.584) tests. The linear regression model showed that the handgrip test was significantly associated with employee vitality levels (β = 0.219; *p* = 0.029), regardless of the covariates included. This model explained 29.9% of the variance in employee vitality scores. The only physical performance test that was associated with workers’ vitality was the handgrip test. Although vitality depends on multiple factors, our findings indicate that companies should promote physical exercise, especially strength training, as an alternative to increase the well-being and health of their employees. 

**Funding:** This research received no external funding.

### 2.20. Dampening and Spring-like Characteristics Differentiating Horizontal Deceleration Ability in Team Sport Athletes

**Damian J. Harper ^1^, Daniel D. Cohen ^2,3^ and Jason S. Pedley ^4^**

^1^ Institute of Coaching and Performance, School of Sport and Health Sciences, University of Central Lancashire, Preston, UK 1; d.harper5@uclan.ac.uk

^2^ Masira Research Institute, Faculty of Health Sciences, University of Santander, Bucaramanga, Colombia 2; danielcohen1971@gmail.com

^3^ Sports Science Centre (CCD), Colombian Ministry of Sport (Mindeporte), Colombia

^4^ Youth Physical Development Centre, Cardiff School of Sport and Health Sciences, Cardiff Metropolitan University, Cardiff, UK 4; jpedley@cardiffmet.ac.uk

A novel method to classify stretch-shortening cycle (SSC) performance using force–time profiles in the drop vertical jump (DVJ) has recently been shown to identify athletes with superior spring-like (i.e., rapid force generation) and force attenuation (i.e., dampening) capabilities [1]. Similar to the DVJ, rapid horizontal decelerations require the generation of high ground reaction forces (GRFs) in short timeframes and could expose athletes to high-impact peaks and loading rates upon ground contact [2]. Therefore, this new DVJ classification approach could provide additional insight into the qualities underpinning rapid horizontal deceleration ability. Twenty-three male (age: 19.9 ± 1.9 years; height 180 ± 8 cm; body mass 74.6 ± 19 kg) team-sport athletes performed (1) DVJs on force plates (PASCO Scientific) connected to ForceDecks acquisition software (Vald Performance) from a 40 cm height and (2) maximal horizontal deceleration, measured using radar (Stalker ATS II), following a 20 m sprint. Using a median split, athletes were dichotomized into high (n = 11; mean = −5.05 ± 0.21 m·s^−2^) and low (n = 12; mean = −4.32 ± 0.42 m·s^−2^) horizontal deceleration, and their SSC function was categorized based on the presentation of a visible impact peak during the first 20% of ground contact, and whether they displayed spring-like behavior (defined as a Pearson product moment correlation between vertical GRF and vertical center-of-mass displacement of <−0.80). Athletes were rated as GOOD (no impact peak and spring-like), MODERATE (impact peak but still spring-like), or POOR (impact peak and not spring-like). Despite similar spring-like behavior, a greater proportion of the high-deceleration group performed the DVJ with no impact peak (82 vs. 33%, respectively). We suggest this combination of features could be due to superior lower limb pre-impact and rapid reactive reflex muscle activation during the downward (eccentric) phase of the jump. During horizontal deceleration, these capabilities may enable higher braking impulses and active force distribution across lower limbs during each braking step, contributing to better horizontal deceleration capacity. 

**Funding:** This research received no external funding 

**References**

1. Pedley, J.S.; Lloyd, R.S.; Read, P.J.; Moore, I.S.; Myer, G.D.; Oliver, J.L. A novel method to categorize stretch-shortening cycle performance across maturity in youth soccer players. *J. Strength Cond. Res.* **2020**. https://doi.org/10.1519/JSC.0000000000003900.
2. Verheul, J.; Nedergaard, N.J.; Pogson, M.; Lisboa, P.; Gregson, W.; Vanrenterghem, J.; Robinson, M.A. Biomechanical loading during running: Can a two mass-spring-damper model be used to evaluate ground reaction forces for high-intensity tasks? *Sports Biomech*. **2021**, *20*, 571–582. https://doi.org/10.1080/14763141.2019.1584238.

### 2.21. Anthropometry, Physiological Markers, and Physical Performance under Control during Amateur Female Soccer Players’ Preseason

**José M. Izquierdo ^1^, Ana M. de Benito ^2^ and Juan C. Redondo ^2^**

^1^ Dpto. Didáctica de la Expresión Musical, Plástica y Corporal, Universidad de Valladolid; josemaria.izquierdo@uva.es 

^2^ Dpto. Educación Física y Deportiva, Universidad de León; ambent@unileon.es; jc.castan@unileon.es

According to the periodization theory, the preseason is the first phase of the training process. Preseason training lasts only 6–8 weeks in professional soccer teams, but it is a crucial period because players often return in a significantly detrained state as a result of the summer intermission period. Adaptations to preseason training stimuli may not occur uniformly in all players. Individual features such as physical fitness, age or body composition, and external conditions (energy-intake, lifestyle habits, main job, etc.) surrounding the players may determine the physiological stress athletes can withstand [1]. That is why it is recommended to assess body composition, physical performance factors, and several biological, hormonal, and psychological markers to periodically monitor both daily training load and fatigue levels [1]. In spite of their practical importance, there is a lack of evidence about preseason changes in physical fitness and physiological markers of amateur female soccer players. Then, the purpose of this study was to investigate the magnitude of changes in anthropometry, sprint and jump capacity, and physiological stress biomarkers among amateur female soccer players during the preseason. Twenty-two amateur female soccer players (23.68 ± 3.69 years) belonging to the same team participated voluntarily in this study. A 5-week training program was arranged for the transition from high-volume and low-intensity workloads to high-intensity and low-volume workloads attending to Matveev periodization. Each week was based on three training sessions (120’ warm-up, physical fitness preparation, and technical and tactical practice) and a friendly match. Body mass (BM), body mass index (BMI), body fat percentage (BF), heart rate variability (HRV) values, logarithm of the root mean square of successive heartbeats interval differences (lnRMSDD), standard deviation of all normal heartbeats intervals (SDNN), high- and low-frequency ratio (LF/HF), salivary cortisol and testosterone concentrations, 20 and 40-m sprint, and countermovement jump were assessed during the program (PRE, weeks 1, 2, 3, 4, and END). Effects related to the week load were assessed using a one-way ANOVA (time) with repeated measures. When Wilks’ Lambda indicated a significant F-value, Scheffe’s post-hoc procedures were performed to determine pairwise differences. Partial eta squared (ƞ^2^p) was computed. Statistical significance was set at *p* < 0.05. Wilks’ Lambda indicated a significant F-value in all variables except LF/LH. In addition, we mainly found differences in the tendency of improved fitness levels after the 5-week preseason training. Scheffe’s post-hoc procedures identified differences between PRE and all measurements in anthropometry variables, and between PRE and END in all variables. Regarding the anthropometric variables, BM, BMI, and BF significantly decreased through all of the preseason’s measurements, likely because female soccer players can accumulate body fat in the off-season and lose weight during the preseason training time. Heart rate variability variables (lnRMSSD and SDNN) demonstrated small decreases in all assessments and showed significant results between PRE vs. END in lnRMSSD; and PRE vs. week 2, 3, 4, and END in SDNN. These changes in lnRMSSD could be interpreted as a positive response due to a concomitant improvement in wellness variables [2]. Furthermore, Flatt et al. [2] found that the lnRMSSDmean measured three days a week is sufficient to register the 7-day average. All this enforces the idea that the implementation of HRV measurement systems in sports teams is convenient. Testosterone and cortisol increased significantly across preseason sessions; however, a decreased T/C ratio was observed, similar to what was reported in previous studies. The ratio decrease might reflect a more catabolic state at the end of the intensified period compared to the beginning of training. Finally, physical capacities improved through the 5-week training program, being significant from PRE to END. These results showed that a lower body fat percentage is associated with better sprint performance. Comotto et al. (2015) stated that acceleration and jump capacities improve from pre-preseason to mid-season due to training loads [3]. Considering that amateur players deal with many external factors, as well as individual features that may contribute to modifying their physiological status, these findings may help coaches and athletes to better understand preseason physiological stress and plan appropriate training strategies to improve athletic performance without risk of injury or overtraining.

**Funding:** This research received no external funding.

### 2.22. Motor Units Activation Changes during Acute Muscle Pain

**Klaus Magno Becker ^1,2^, Marcio Goethel ^1,2^, Manoela Sousa ^1,2^, João Paulo Vilas Boas ^1,2^ and Ulysses Ervilha ^3^**

^1^ Center of Research, Education, Innovation and Intervention in Sport (CIFI2D), Faculty of Sport, University of Porto, Porto, Portugal; klausmagnobecker@gmail.com (K.M.B.); gbiomech@fade.up.pt (M.G.); manoelavsousa@fade.up.pt (M.V.S.); jpvb@fade.up.pt (J.P.V.B.)

^2^ Porto Biomechanics Laboratory, Faculty of Sport, University of Porto (LABIOMEP), Porto, Portugal

^3^ School of Arts, Sciences and Humanities, University of São Paulo, São Paulo, Brazil; ulyervil@usp.br

It is not fully understood how muscles can produce the same level of force under pain compared to non-pain conditions [1]. In order to investigate this phenomenon, it is necessary to assess the behavior of Motor Units (MU), which is possible through the decomposed electromyography signal [2]. This study aimed to compare the effects of induced muscle pain on the MU firing rate (FR) and recruitment threshold (RT), which is the value in V needed to recruit MUs. Based on MUs’ structural differences, the use of the silhouette criterion [3,4] approach allowed us to access the optimal number of clusters that can be found in the full set of MUs. Then, using a Kmeans approach, the MUs were grouped in clusters. A visual analog scale of 10 cm was used to quantify pain during the session, in which 0 cm represents the absence of pain and 10 cm represents the maximum level of pain. Six volunteers participated in this study. Before the beginning of the task, electrodes (Galileo-DelsysTM) were positioned in vastus lateralis and rectus femoris muscles. The task was performed using an isokinetic dynamometer (BiodexTM system 4) and consisted of two MVICs and, later, five isokinetic maximum concentric/eccentric contractions at 60°/s for knee extensors. All volunteers were submitted to a single bolos, one week apart, of 2.0 mL of intramuscular hypertonic 6% (Hyper) or isotonic 0.9% (Iso) saline solution, which were applied to induce pain or the placebo, respectively. Immediately after the infusion, they repeated the described protocol. A MANOVA test was used to analyze FR and RT variables between clusters, moments, and conditions, with a significance level of 0.05 and Bonferroni correction in the pairwise. The visual analog scale responses after infusions were Iso (pre = 0 and post = 1.8 ± 2.1) and Hyper (pre = 0 and post = 6.5 ± 2.7), and a difference was found between conditions (*p* < 0.001). The Kmeans distinguished all MUs in two groups (G1 and G2) that showed differences in all moments and conditions (*p* < 0.001). Regarding FR, in G1 of the MUs condition Iso and moment pre vs. Iso post, there was an increase (*p* = 0.03), whilst Hyper pre vs. post decreased (9.2 ± 2.6 to 2.6 ± 1.6, *p* < 0.001). Iso post vs. Hyper post also presented a difference (*p* < 0.001), and this comparison disregards the effect of intramuscular injection. The MUs of G2 decreases the FR on Hyper pre vs. post (2.9 ± 1.6 to 8.4 ± 2.5) and Iso post vs. Hyper post comparisons (*p* < 0.001). The RT in G1 increases in Hyper pre × post (0.19 V ± 0.09 V to 0.46 V to 0.3 V) and Iso post vs. Hyper post G1 (*p* < 0.001), and G2 did not change the RT. However, both G1 and G2 separately show difference in pre vs. pre (*p* < 0.001). Current data show that the strategy for maintaining force during pain is to increase the FR of motor units with lesser FR already recruited without pain and preserve the preferential recruited motor unit of ordinarily higher FR.

**Funding:** This research received no external funding.

**References**

1. Hodges, P.W.; Tucker, K. Moving differently in pain: A new theory to explain the adaptation to pain. *Pain* **2011**, *152* (Suppl. 3), S90–S98. 10.1016/j.pain.2010.10.020.
2. De Luca, C.J; Adam, A.; Wotiz, R.; Gilmore, L.D.; Nawab, S.H. Decomposition of Surface EMG Signals. *J. Neurophysiol.* **2006**, *96*, 1646–1657. 10.1152/jn.00009.2006.
3. Rouseeuw, P.J. Silhouettes: A graphical aid to the interpretation and validation of cluster analysis. *J. Comput. Appl. Math.* **1987**, *20*, 53–65. https://doi.org/10.1016/0377-0427(87)90125-7.
4. Arthur, D.; Sergi V. K-means++: The Advantages of Careful Seeding. In Proceedings of the SODA ‘07: Proceedings of the Eighteenth Annual ACM-SIAM Symposium on Discrete Algorithms, New Orleans LA, USA, 7–9 January 2007; pp. 1027–1035.

### 2.23. Short-Term Effects of Dynamic Neuromuscular Stabilization on Postural Stability and Mobility in Rowers

Dragan Marinkovic ^1,^*, Dejan Madic ^1^, Drazenka Macak ^1^, Danilo Radanovic ^1^, Zoran Gojkovic ^2^, Boris Popovic ^1^ and Nebojsa Trajkovic ^3^

^1^ Faculty of Sport and Physical Education, University of NoviSad, 21000 NoviSad, Serbia; marinkovic@uns.ac.rs (D.M.); dekimadic@gmail.com (D.M.M.); macak.md@yahoo.com (D.M.); radanilo17@yahoo.com (D.R.); borispopovic0803@gmail.com (B.P.)

^2^ Provincial Government and Provincial Secretary for Healthcare, 21000 NoviSad, Serbia; zoran.gojkovic@vojvodina.gov.rs

^3^ Faculty of Sport and Physical Education, University of Niš, 18000 Niš, Serbia; nele_trajce@yahoo.com

* Correspondence: D.M.marinkovic@uns.ac.rs

It is well known that good postural stability and trunk flexibility are among the most important prerequisites for enhancing sports performance and achieving superior results [1,2,3]. In rowing, one or several athletes in a sitting position propel the boat through water via cyclical manipulation, which renders their posture unstable [4]. Consequently, adequate trunk flexibility and center-of-mass control are highly important, as biomechanical stability is essential for balance maintenance in all equestrian sports requiring prolonged sitting [5]. While different training modalities have been shown to improve those parameters, Dynamic Neuromuscular Stabilization (DNS) is particularly effective for enhancing postural stability in sport-specific environments. The aim was to explore acute effects of DNS on postural stability and trunk flexibility in elite junior rowers. The study sample comprised 40 junior rowers (16 girls and 24 boys; age 17.4 ± 2.6 years; BMI 22.3 ± 2.4 kg/m^2^). As a part of the study, the participants engaged in a 7-day DNS training regimen involving 2 × 30 min of daily DNS core exercise protocols as part of an international rowing camp. Testing was performed after the first day of familiarization and at the end of the training camp (at a 7-day time point). For this purpose, laboratory-grade force plate (Footscan^®^ plate; RSscan International, Beringen, Belgium) measurements were conducted, and the data weere used to determine postural stability during the 30 s double-leg stand upright quietly with eyes open test, as well as the sit and reach test, commonly employed to assess the flexibility of the lower back and hamstring muscles. Postural stability was quantified in terms of area, anterior–posterior displacement (AP), medial–lateral displacement (ML), and displacements of the center of force (COF) as well as length function of surface (LFS). Trunk flexibility was evaluated through the sit and reach test. Paired *t*-tests revealed that, after a short-term DNS training intervention, all parameters related to postural stability and trunk flexibility were improved in all elite junior rowers (*p* < 0.001). On average, COF declined by −0.94 cm (95% CI [−1.09, −0.78], LFS was reduced by −0.06 cm (95% CI [−0.10, −0.03]), AP by −0.79 cm (95% CI [−1.00, −0.60]), and ML by −0.37 cm (95% CI [−0.47, −0.26]). Moreover, the sit and reach test mean performance improved by an average of 2.37 cm (95% CI [1.72, 3.01]). Our results suggest that a 7-day DNS training program yields improvements in postural stability and trunk flexibility. DNS training sessions appear to be sufficient at inducing positive changes in neuromuscular core anticipation and flexibility, allowing athletes to perform well during training or competition. In summary, the DNS protocol employed in this study is a promising new approach to improving postural stability and trunk flexibility in rowing and should be considered by coaches and experts in their training practice.

**Funding:** This research received no external funding.

**References**

1. Liang, Y.; Hiley, M.; Kanosue, K. The effect of contact sport performance level on postural control. *PLoS ONE* **2019**, *14*, e0212334.
2. Andreeva, A.; Melnikov, A.; Skvortsov, D.; Akhmerova, K.; Vavaev, A.; Golov, A.; Draugelite, V.; Nikolaev, R.; Chechelnickaia, S.; Zhuk, D.; et al. Postural stability in athletes: The role of age, sex, performance level, and athlete shoe features. *Sports* **2020**, *8*, 89.
3. Hrysomallis, C. Balance ability and athletic performance. *Sports Med.* **2011**, *41*, 221–232.
4. Chung, H.C. The influence of kayaking and rowing sports experience on postural response to optic flow. *Percept. Mot. Skills* **2015**, *120*, 1–14.
5. Marinkovic, D.; Pavlovic S.; Madic, D.; Obradovic, B.; Nemeth Z.; Belic, A. Postural stability—A comparison between rowers and field sport athletes. *JPES* **2021**, *21*, 1525–1532.

### 2.24. Biomechanical Evaluation of Posture in Children That Do and Do Not Partake in Sports Activities

Dragan Marinkovic ^1,^*, Dejan Madic ^1^, Drazenka Macak ^1^, Danilo Radanovic ^1^, Aleksandra Ilic ^1^, Zoran Gojkovic ^2^ and Boris Popovic ^1^

^1^ Faculty of Sport and Physical Education, University of Novi Sad, 21000 Novi Sad, Serbia; marinkovic@uns.ac.rs (D.M.); dekimadic@gmail.com (D.M.M.); macak.md@yahoo.com (D.M.); radanilo17@yahoo.com (D.R.); play.dance.studio@gmail.com (A.I.);borispopovic0803@gmail.com (B.P.)

^2^ Provincial Government and Provincial Secretary for Healthcare, 21000 Novi Sad, Serbia; zoran.gojkovic@vojvodina.gov.rs

* Correspondence: D.M.marinkovic@uns.ac.rs

Available scientific evidence indicates that poor posture is often formed in childhood and can, over time, progress to different deformities identified in adults [1,2,3]. However, engagement in sports activities and regular exercise, as well as a healthy lifestyle, promotes movement, muscle activation, and the formation of correct posture in childhood, thereby increasing the likelihood that an individual would maintain good posture [4]. To evaluate and compare posture in children with different levels of involvement in sports activities, the study sample comprised 290 children (114 girls and 176 boys; age 6.83 ± 1.8 years; BMI 15.33 ± 1.13 kg/m^2^), 189 of whom regularly participated in a variety of sports activities, and the remaining 101 did not. Using the 3D Photometric Contemplas system (Templo^®^ Posture Analysis), the following biomechanical posture parameters were measured for both groups: Shoulder rotation (SR) and Pelvic rotation (PR) in the horizontal plane; Distance cervical spine–sacrum (APc), Distance thoracic spine–sacrum (Apt), and Distance lumbar spine–sacrum (APl) in the sagittal plane; and Distance cervical spine–sacrum (MLc), Distance thoracic spine–sacrum (MLt), and Distance lumbar spine–sacrum (MLl) in the frontal plane. Digital calculations were performed for distance, angles, and displacement. A multivariate analysis of variance showed that 67.2% of the variance in biomechanical posture parameters was associated with sports participation (*p* < 0.001). Moreover, on average, with the exception of APc, APl, and MLc, the values of studied biomechanical posture parameters were significantly lower in children that actively engaged in sports (*p* < 0.05), as determined by one-way analysis of variance. Their mean SR and PR in the horizontal plane was also greater by 4.80° (95% CI = 4.21–5.91) and 4.70° (95% CI = 4.03−5.29), respectively, relative to the non-sports group, while their mean APt in the sagittal plane, as well as MLt and MLs in the frontal plane, was lower by 0.33 cm (95% CI = 0.08–0.59), 0.26 cm (95% CI = 0.10–0.42), and 0.70 cm (95% CI = 0.01–0.14), respectively. The findings yielded by the present study suggest that involvement in diverse sports during childhood improves the biomechanical parameters related to posture, providing support for available evidence indicating that exercise programs can lessen the extent of different postural disorders in children. Specifically, the reported results imply that kyphosis, lordosis, and scoliosis and other postural issues are less prevalent in children that are physically active relative to those that are not. Sports and other forms of physical exercise should be used as corrective and preventive treatment in childhood to enhance muscle activation and inhibit postural deformities in adulthood.

**Funding:** This research received funding from IPA Cross Border programme Serbia and Bosnia and Herzegovina “Improving testing abilities on pos-tural and spinal column status—SpinLab”, founded by the European Union, Directorate for European Integration (EU-IPA 2013/323-164).

**References**

1. Ćirić, A.; Čaušević, D.; Bejdić, A. Differences in posture status between boys and girls 6 to 9 years of age. *Homosporticus* **2015**, *17*, 15.
2. Kapo, S.; Rađo, I.; Smajlović, N.; Kovač, S.; Talović, M.; Doder, I.; Čović, N. Increasing postural deformity trends and body mass index analysis in school-age children. *Zdr. Varst*. **2018**, *57*, 25–32.
3. Kovac, S.; Kajmovic, H.; Radjo, I.; Manic, G. Trend projections of body deformities occurrence between the ages of 5 and 12, metrically objectified and estimated by 3D postural status screening. *Homosporticus* **2014**, *17*, 5–14.
4. Molina-Garcia, P.; Mora-Gonzalez, J.; Migueles, J.H.; Rodriguez-Ayllon, M.; Esteban-Cornejo, I.; Cadenas-Sanchez, C.; Plaza-Florido, A.; Gil-Cosano, J.J.; Pelaez-Perez, M.A.; Garcia-Delgado, G.; et al. Effects of exercise on body posture, functional movement, and physical fitness in children with overweight/obesity. *J. Strength Cond. Res*. 2020, *34*, 2146–2155.

### 2.25. Frequency of Eccentric-Based Exercises during the Competitive Microcycle: A Survey of Strength and Conditioning Coaches of Elite European Soccer Teams

**Antonio Martínez-Serrano ^1^, Pedro Gómez-Piqueras ^2^ and Pedro E. Alcaraz ^1^**

^1^ UCAM Research Center for High Performance Sport, Catholic University of Murcia, 30107 Murcia, Spain; amartinez30@ucam.edu; palcaraz@ucam.edu

^2^ Department of Physical Education, University Castilla La Mancha, Albacete, Spain; dr.pedro.gomez.piqueras@gmail.com

One of the main concerns of modern soccer teams is to avoid injuries in order to achieve the highest player availability in training sessions and competitive matches. Although injury incidence seems to have decreased in recent years, this trend has not changed in muscle-type injuries [1], frequently located in the hamstring muscles. Therefore, most teams have developed specific injury prevention programs, mostly based on eccentric (ECC) exercises (i.e., nordics, deadlift, leg curls, etc.), to minimize the risk of hamstring strain injuries. However, if the scheduling of ECC-based exercises during the microcycle is not properly performed, it could impair match performance or increase injury risk [2], since the effects of ECC training (i.e., muscle damage, weakness, or soreness) can last up to 48–72 h [3]. Therefore, the purpose of this study was to analyze how frequently strength and conditioning coaches (SCCs) from professional European teams program ECC-based resistance exercises during the competitive microcycle. An observational survey design on a specific cohort was used. In total, 201 European first- and second-division teams from different countries were invited to participate in this study. Forty-two SCCs (age = 36.8 ± 7.2 years; experience = 9.7 ± 6.3 years) accepted the invitation and completed the survey. The survey consisted of three types of ECC tasks (hip-dominant eccentric exercises (H-ECC), knee-dominant eccentric exercises (K-ECC), and high-load eccentric exercises with external devices (Ext-ECC)). SCCs coaches had to report how often (never (N), sometimes (S), frequently (F), or always (A)) they performed these tasks on each of the days of the competitive microcycle (MD+1, MD+2, MD-4, MD-3, MD-2, MD-1). A frequency analysis and the χ^2^ goodness of fit test were performed. On MD-4, the majority of SCCs frequently used H-ECC (23 [54.8%]; *p* ≤ 0.001; χ^2^ = 26.381) and K-ECC (19 [45.2%]; *p* = 0.022; χ^2^ = 9.619), and sometimes Ext-ECC (17 [40.5%]; *p* = 0.011; χ^2^ = 11.143). However, there was no agreement on how frequently they performed H-ECC (*p* = 0.655; χ^2^ = 1.619) and K-ECC (*p* = 0.251; χ^2^ = 4.095) on MD-3, although most of them never used Ext-ECC (21 [50.0%]; *p* ≤ 0.001; χ^2^ = 18.762). In conclusion, SCCs from professional European teams mainly performed ECC-based exercises on MD-4 and MD-3. From a practical perspective, SCCs should be aware of potential detrimental effects in match performance when conducting high-intensity ECC training in the hamstring muscles on MD-3.

**Funding:** This research received no external funding.

**References**

1. Ekstrand, J.; Spreco, A.; Bengtsson, H.; Bahr, R. Injury rates decreased in men’s professional foot-ball: An 18-year prospective cohort study of almost 12 000 injuries sustained during 1.8 million hours of play. *Br. J. Sports Med*. **2021**, *55*, 1084-1091. https://doi.org/10.1136/bjsports-2020-103159.
2. Douglas, J.; Pearson, S.; Ross, A.; McGuigan, M. Chronic Adaptations to Eccentric Training: A Systematic Review. *Sports Med.* **2017**, *47*, 917–941, https://doi.org/10.1007/s40279-016-0628-4.
3. Nassis, G.P.; Brito, J.; Figueiredo, P.; Gabbett, T.J. Injury prevention training in football: Let’s bring it to the real world. *Br. J. Sports Med*. **2019**, *53*, 1328, https://doi.org/10.1136/bjsports-2018-100262.

### 2.26. Will Core Stability Training Affect Performance in Surfers?

**Vít Musil ^1,2,3^, João Paulo Vilas-Boas ^2^ and Márcio Borgonovo-Santos ^2,3^**

^1^ Faculty of Physical Culture, Palacký University, Olomouc, Czech Republic; vita.musil@gmail.com

^2^ Centre of Research, Education, Innovation and Intervention in Sport (CIFI2D), Faculty of Sport and Porto Biomechanics; jpvb@fade.up.pt

^3^ Riedel Communication GmbH & Co. KG, R&D Hub Portugal; Marcio.Santos@riedel.net

The science applied to surfing faces narrow participation of physiotherapy [1,2], despite the possibility of proposing new insight into current knowledge, and thus enriching sports science via a multidisciplinary approach [3]. This work aimed to develop a core stability training program and verify its potential efficiency using a sprint paddling-specific test battery (TB) [4]. A field experiment with an exploratory design was applied to the group of 15 surfers: 10 males (age: 29.1 ± 5.9 years, weight: 77.2 ± 8.5 kg, height: 181.2 ± 8.1 cm) and 5 females (age: 28.4 ± 4.6 years, weight: 61.8 ± 3.1 kg, height: 165.6 ± 2.9 cm). TB was utilized to collect data before the initiation of training and consisted of five consecutive tests related to the demands of sprint paddling [4]. A seated medicine ball throw (SMBT) analyzed arm pushing power, the one-leg stand test (OLST) examined postural control, the trunk lift test (TLT) assessed back strength and flexibility, closed kinetic chain upper extremity stability (CKCUEST) focused on the stability of the upper body, and the sit-ups test (SUT) evaluated abdominal strength and power. The 4-week intervention of two dependent exercise units was subsequently applied. Within the first exercise unit, the surfers performed isometrical contraction of core stabilizers to maintain the position, while the second contained whole-body movements with an emphasis on precise execution. The inclusion of breathing techniques was targeted to enhance diaphragm activation. The subjects were led by a physiotherapist individually or in a group with a maximum of four people. Initially, the body was expected to adapt to a new stimulus and subsequently increase muscle capacity over the following sessions by applying resistance against movement or strict control of the exercise position. At the end of the intervention, each participant underwent a final evaluation and received an evaluation protocol. For the entire period, the International Physical Activity Questionnaire (IPAQ) was used as a variable to monitor movements [5]. Descriptive statistics and correlation were used to describe the results. The best of three trials was selected for each test. The horizontal distance of SMBT (PRE: 568 ± 143.4 cm, POST 592 ± 128 cm), vertical measure of TLT (PRE: 28 ± 6.2 cm, POST: 30 ± 7.9 cm), repetitions of CKCUEST (PRE 26 ± 3.4, POST 31 ± 4.5), as well as SUT (PRE 24 ± 2.2, POST 26 ± 4.2) increased. The entire group reached the maximum in OLST. The correlation values for the comparison of differences of max. PRE, POST trial, and total MET/min/month, except the OLST, are as follows: (rSMBT = 0.13, rTLT = 0.49, rCKCUEST = −0.36, rSUT = 0.5, rmean = 0.19). Superior performance post-evaluation suggests the effectiveness of the physiotherapy-based training protocol. The authors suggest assessing sprint paddling in water to support decision-making on performance indicators and evaluating the intervention’s effect from the perspective of a kinematic analysis. Lastly, our findings suggest the replacement of OLST by the more appropriate tool.

**Funding:** This research received no external funding.

**References**

1. Donaldson, T.; Scantlebury, M.; Furness, J.; Kemp-Smith, K.; Newcomer, S.; Climstein, M. Training Methods in the Sport of Surfing: A Scoping Review. *Strength Cond. J.* **2021**, *publish ahead of print*.
2. McArthur, K.; Jorgensen, D.; Climstein, M.; Furness, J. Epidemiology of Acute Injuries in Surfing: Type, Location, Mechanism, Severity, and Incidence: A Systematic Review. *Sports* **2020**, *8*, 25
3. Davidek, P.; Andel, R.; Kobesova, A. Influence of Dynamic Neuromuscular Stabilization Approach on Maximum Kayak Paddling Force. *J. Hum. Kinet.* **2018**, *61*, 15–27.
4. Minghelli, B.; Paulino, S.; Graça, S.; Sousa, I.; Minghelli, P. Time-Motion Analysis of Competitive Surfers: Portuguese championship. *Rev. Assoc. Med. Bras.* **2019**, *65*, 810–817.
5. International Physical Activity Questionnaire. Available online: http://www.ipaq.ki.se/scoring.pdf (accessed on 1 March 2021).

### 2.27. On the Need to Measure Ball Speed and Ball Hitting Precision in Soccer: A Critical Review

**Martín O’Justo ^1,2^, Dan Río-Rodríguez ^1,2^ and Rafael Martín-Acero ^1,2^**

^1^ Grupo de Aprendizaje y Control del Movimiento Humano, Facultade de Ciencias do Deporte e a Educación Física, Universidade da Coruña, Avenida Ernesto Che Guevara 174, Pazos Lians 15179 Oleiros (A Coruña), Spain

^2^ ATP Entrenamiento Personal, Rúa Ícaro 2ª, 15179, Oleiros, A Coruña, Spain

In the scientific literature, there is a gap in the investigations of the shot on goal, as the speed of the ball and the precision of the shot at goal are not considered simultaneously, and when they have been studied in soccer players of formative ages, speed and precision have been separated. We have reviewed 59 articles related to the accuracy and power of shooting in soccer. Heterogeneous proposals have been found. To our knowledge, there is no validity test to measure the accuracy of shooting in soccer. The purpose of the review was to analyze what had been measured and how it had been measured to make a proposal consistent with the objectives of our study and the state of the topic in question. For this reason, we have performed a thorough analysis of the criteria related to accuracy and shooting in each scientific article: The number of sessions, the sample of studies, years of experience of the players, competitive level, the shooting surface, the shot in relation to the specific position, the competitive time in which the testing was carried out, the dominant and non-dominant leg, instruments for measuring and recording, the statics or dynamics of the ball when hit, the distance between the ball and the goal, the type of shot taken, the approach to the ball, the distance between the shooter and the ball, the number of steps that are allowed prior to shooting, the angle allowed for shooting, measurement practice prior to shooting, the number of shots taken in the test, and the types of goal-targets to be hit. Some studies have shown that hitting with precision demand is not an age-dependent ability, since no change with age has been found. However, in a recent study (1), the hitting precision parameters seemed to be damaged in adolescence, near the period of accelerated growth between 13 and 15 years (individually, when their PHV is equal to 0). On the contrary, they found that the Vball increased linearly, as age increased. Considering our latest study, where the transversal evolution of the ability to hit with both legs, conducted in R.C. Deportivo de La Coruña, shows that the maximum speed of the ball improves with age, and notably from U-11 to U-15 and much more slowly thereafter, it seems that having continuous improvement of neuromuscular capacity, as well that in the players of the Vieira et al. [1], it is necessary to study the loss of hitting precision also described by Vieira et al. [1]. In the same study, it can be counteracted by training before and during the so-called “body growth crisis”. A longitudinal study is required on the mutual affectation of the increase in muscular power and the evolution of hitting precision in the ages with the highest rate of body growth in male and female soccer players. Our proposal to measure the precision in soccer shooting is based on the attempt to find the possible curve between precision and power. This relationship between precision and power would be established by the degree of success achieved at each intensity (ball velocity) of the shots made. It would be interesting to carry out a universal design to perform measurements in samples of different ages, sex, laterality, and competitive level.

**Funding:** This research received no external funding.

**Reference**

1. Vieira, L.H.; Cunha, S.A.; Moraes, R.; Barbieri, F.A.; Aquino, R.; Oliveira, L.D.P.; Navarro, M.; Bedo, B.L.; Santiago, P.R. Kicking Performance in Young U9 to U20 Soccer Players: Assessment of Velocity and Accuracy Simultaneously. *Res. Q. Exerc. Sport* 2018, *89*, 210–220. https://doi.org/10.1080/02701367.2018.1439569.

### 2.28. Curve Sprinting Effects on Spatiotemporal Parameter Symmetry: A Systematic Review

**Elí O. Paz-Paz, Pedro Peréz-Soriano and Alberto Encarnación-Mártinez**

Research Group in Sports Biomechanics (GIBD), Departament of Physical Education and Sports, University of Valencia, 46010 Valencia, Spain; epazpaz@alumni.uv.es

In total, 50% of the sprint in 200 and 400 m races in athletics is performed on a curve [1]. It has been shown that, during curve sprints, the velocity decreases compared to sprints in a straight line [2,3,4] (proportional effects according to the radius) [5,6]. Therefore, it has been suggested that athletes in internal lanes could have a biomechanical disadvantage [5]. We aimed to describe tee possible differences between the leg distribution in spatiotemporal parameters when curve sprinting. A systematic review, following the basis of the PRISMA statement [7], was carried out using PubMed, SPORTDiscus with full texts, and MEDLINE databases. Studies published from 1 January 1951 to 14 April 2020 were searched. After analyzing the titles and abstracts to identify the study’s relevance, the inclusion criteria were studies (1) evaluating healthy elite athletes (regardless of gender), (2) reporting athletic anthropometric characteristics, (3) including sprinters from 200/400 m outdoor tracks, (4) with details of the radius/lane (Track approved IAAF), and (5) focused on analyze spatiotemporal parameters. Duplicate studies were eliminated, and potential articles were stored using the Mendeley tool. Studies’ methodological quality and qualitative synthesis were evaluated [8]. All studies [5,9,10,11,12,13] received a high rating in general and external evaluations, four studies [5,9,10,11] received a high rating in internal evaluation, and two studies [12,13] received a good rating. In total, 907 studies were found, but only 6 fulfilled the inclusion criteria [5,9,10,11,12,13]. These studies analyzed races on an external track curve in at least one radius/track with elite athletes. Analyses were reported in five different radii and only one study [12] reported significant differences in velocity. Two studies [5,12] reported step frequency and step length, one reported flight time [12], and five studies [5,9,10,11,12] reported significant differences in ground contact time in lanes one, two, four, five, and eight (radius 36.5, 37.72, 41.41, 45.10). Ground contact time was greater for the inside leg in seven different study conditions [5,9,10,11,12]. Only one study [5] showed significant differences between lanes two, four, and eight (radius 37.72, 41.41 and 45.10) for ground contact time, showing greater times for the internal lanes in both legs. The ground contact time was longer for the inside leg, which suggests a specific function for each leg. The inside leg supports a greater mass on the sprint, so it requires greater power generation. However, the outside leg is responsible for generating stability and orientation. An interaction between the lane and the biomechanical response cannot be conclusively defined, but it suggests longer ground contact time in the internal lanes (but only one study demonstrates it) [5]. No relevant results were shown in velocity, step frequency, step length, and flight time.

**Funding:** This research received no external funding.

**References**

1. IAAF. Reglamento de Competición. International Association of Athletics Federations. 2018. Available online: https://www.iaaf.org/about-iaaf/documents/rules-regulations (accessed on 10 March 2021).
2. Jain, P.C. On a discrepancy in track races. *Res. Q. Exerc. Sport*. **1980**, *51*, 432–436. https://doi.org/10.1080/02701367.1980.10605212.
3. Quinn, M.D. The effect of track geometry on 200- and 400-m sprint running performance. *J. Sports Sci*. **2009**, *27*, 19–25. https://doi.org/10.1080/02640410802392707.
4. Greene PR. Running on flat turns: Experiments, theory, and applications. *J. Biomech. Eng*. **1985**, *107*, 96–103. https://doi.org/10.1115/1.3138542.
5. Churchill, S.M.; Trewartha, G.; Salo, A.I.T. Bend sprinting performance: New insights into the effect of running lane. *Sports Biomech.* **2019**, *18*, 437–447. https://doi.org/10.1080/14763141.2018.1427279.
6. Ferro, A.; Floria, P. Differences in 200-M sprint running performance between outdoor and indoor venues. *J. Strength Cond. Res.* **2013**, *27*, 83–88. https://doi.org/10.1519/JSC.0b013e31824f21c6.
7. Urrútia, G.; Bonfill, X. Declaración PRISMA: Una propuesta para mejorar la publicación de evisions sistemáticas y metaanálisis. *Med. Clin. (Barc.)* **2010**, *135*, 507–511. https://doi.org/10.1016/j.medcli.2010.01.015.
8. Berra, S.; Elorza Ricart, J.M.; Estrada, M.D.; Sánchez, E. Instrumento para la lectura crítica y la evaluación de estudios epidemiológicos transversales. *Gac. Sanit*. **2008**, *22*, 492–497. https://doi.org/10.1157/13126932.
9. Alt, T.; Heinrich, K.; Funken, J.; Potthast, W. Lower extremity kinematics of athletics curve sprinting. *J Sports Sci*. **2015**, *33*, 552–560. https://doi.org/10.1080/02640414.2014.960881.
10. Churchill, S.M.; Trewartha, G.; Bezodis, I.N.; Salo, A.I.T. Force production during maximal effort bend sprinting: Theory vs. reality. *Scand. J. Med. Sci. Sports* **2015**, *26*, 1171–1179. https://doi.org/10.1111/sms.12559.
11. Churchill, S.M.; Salo, A.I.T.; Trewartha, G. The effect of the bend on technique and performance during maximal effort sprinting. *Sports Biomech*. **2015**, *14*, 106–121. https://doi.org/10.1080/14763141.2015.1024717
12. Ishimura, K.; Sakurai, S. Asymmetry in Determinants of Running Speed During Curved Sprinting. *J. Appl. Biomech*. **2016**, *32*, 394–400. https://doi.org/10.1123/jab.2015-0127.
13. Ohnuma, H.; Tachi, M.; Kumano, A.; Hirano, Y. How to Maintain Maximal Straight Path Running Speed on a Curved Path in Sprint Events. *J. Hum. Kinet*. **2018**, *62*, 23–31. https://doi.org/10.1515/hukin-2017-0175.

### 2.29. Effects of Repeated Sprinting on the Hamstring Stiffness Pattern and Neuromuscular Parameters

Ricardo Pimenta ^1,^*, Tomás Lopes ^2^ and José Correia ^2^

^1^ CIPER, Faculdade de Motricidade Humana, Universiade de Lisboa, Cruz Quebrada Dafundo, Portugal; rjl.pimenta@gmail.com*; jpcorreia.ft@gmail.com

^2^ King’s College London, Department of Biochemistry; lopestomas2002@gmail.com

The purpose of this study was to examine the acute effects of a repeated sprint protocol (i) on neuromuscular parameters, (ii) the hamstring stiffness pattern, and (iii) to test the hamstring stiffness pattern between-day repeatability in two sessions separated by 7 days. Eighteen males without a history of hamstring strain injury (HSI) participated in each session. Muscle stiffness was assessed using ultrasound-based shear wave elastography at rest and during isometric knee flexion at 20% of maximal voluntary contraction before and after a 10 × 30 m repeated sprint protocol. There was a significant decrease in speed of 9.4% between the fastest and slowest sprints (*p* < 0.001; *d* = 1.01) with moderate between-day repeatability of the fastest sprint speed (ICC = 0.54). Concerning the neuromuscular parameters, a significant pre–post decrease was observed in peak torque (PT, 6.1% to 10.7%), the rate of torque development (RTD) 0–50 ms (12.5% to 23.1%), and RTD 50–100 ms (14.5% to 19.7%), for both sessions and in both limbs (PT: *p* < 0.001 to 0.048; RTD 0–50 ms: *p* = 0.003 to 0.022; RTD 50–100 ms: *p* < 0.001 to 0.002), with between-day repeatability ranging from poor to good among all neuromuscular parameters (ICC = 0.42–0.89). A decrease of 5.8% in passive stiffness was observed after the task for the biceps femoris long head (BFlh) (*p* = 0.031; η^2^_p_ = 0.246), 8.7% for the biceps femoris short head (*p* = 0.021; η^2^_p_ = 0.275), and 10.9% for the semitendinosus (*p* < 0.001; η^2^_p_ = 0.654), with between-day repeatability ranging between poor and good across muscles (ICC = 0.13–0.85). Regarding active stiffness, only a significant increase of 7.9% was observed in the BFlh (*p* = 0.032; η^2^_p_ = 0.242) after the sprint task. The between-day repeatability of active stiffness measurement was moderate across muscles (ICC = 0.50–0.73). Differences in both the passive and active stiffness of individual muscles were within the standard error of measurement; thus, care is warranted when interpreting these findings. As for the biceps femoris long head/hamstring medial stiffness (BFlh/MH) ratio, an increase of 10% was seen after the fatigue task (*p* = 0.048; η^2^_p_ = 0.212). The present study provides evidence that repeated sprinting impacts speed performance and neuromuscular parameters such as PT and early RTD (0–50 ms; 50–100 ms) without physiologically impacting individual contributions of muscle stiffness in individuals without a history of HSI. However, it should be noted that the increase in the BFlh/MH ratio is an indicator of a load sharing pattern; moreover, the greater contribution of BFlh has also been reported in previous studies on knee flexion task until exhaustion [1,2] and could explain why this muscle has a greater injury occurrence [3,4]. Between-day repeatability was moderate for speed, major neuromuscular parameters, and active stiffness; however, passive stiffness repeatability was low.

**Funding:** The author Ricardo Pimenta is funded by a scholarship from the University of Lisbon/Faculty of Human Kinetics (number 793) and José Correia is funded by a scholarship from Fundação para a Ciência e Tecnologia (number: SFRH/BD/143927/2019).

**References**

1. Mendes, B.; Firmino, T.; Oliveira, R.; Neto, T.; Cruz-Montecinos, C.; Cerda, M.; Correia, J.P.; Vaz, J.R.; Freitas, S.R. Effects of Knee Flexor Submaximal Isometric Contraction until Exhaustion on Semitendinosus and Biceps Femoris Long Head Shear Modulus in Healthy Individuals. *Sci. Rep.* **2020**, *10*, 16433. https://doi.org/10.1038/s41598-020-73433-1
2. Schuermans, J.; Van Tiggelen, D.; Danneels, L.; Witvrouw, E. Biceps Femoris and Semitendinosus—teammates or Competitors? New Insights into Hamstring Injury Mechanisms in Male Football Players: A Muscle Functional MRI Study. *Br. J. Sports Med.* **2014**, *48*, 1599–1606. https://doi.org/10.1136/bjsports-2014-094017
3. Ekstrand, J.; Lee, J.C.; Healy, J.C. MRI Findings and Return to Play in Football: A Prospective Analysis of 255 Hamstring Injuries in the UEFA Elite Club Injury Study. *Br. J. Sports Med.* **2016**, *50*, 738–743. https://doi.org/10.1136/bjsports-2016-095974
4. Opar, D.A.; Williams, M.D.; Shield, A.J. Hamstring Strain Injuries. *Sports Med.* **2012**, *42*, 209–226. https://doi.org/10.2165/11594800-000000000-00000.

### 2.30. Association between Excess Weight and Trunk Muscle Strength

Ana Resende-Coelho ^1,2,^*, Florêncio Diniz-Sousa ^1,2^, Lucas Veras ^1,2^, Giorjines Boppre ^1,2^, Edgar Moutinho-Ribeiro ^1,2^, Vítor Devezas ^3^, Hugo Santos-Sousa ^3^, John Preto^3^, Leandro Machado ^4,5,^ João P. Vilas-Boas ^4,5^, José Oliveira ^1,2^ and Hélder Fonseca ^1,2^

^1^ Research Centre in Physical Activity, Health and Leisure (CIAFEL), Faculty of Sport, University of Porto, Porto, Portugal

^2^ Laboratory for Integrative and Translational Research in Population Health (ITR), University of Porto, Porto, Portugal

^3^ Centro de Responsabilidade Integrado de Obesidade (CRIO), Centro Hospitalar Universitário de São João, Porto, Portugal

^4^ Centre of Research, Education, Innovation and Intervention in Sport (CIFI2D), Faculty of Sport, University of Porto, Porto, Portugal

^5^ Porto Biomechanics Laboratory (LABIOMEP), University of Porto, Porto, Portugal

* anccatarina@gmail.com

Obesity is commonly associated with lower physical function [1,2]; however, its impact on muscle strength, particularly at the trunk, remains unclear. We aimed to investigate the relationship between obesity severity and trunk muscle strength. A total of 154 subjects (59% females), aged 35.3 ± 12.3 years with a body mass index (BMI) of 35 ± 10.5 kg·m^−2^, were recruited. Body composition (dual-energy X-ray absorptiometry) and anthropometry were assessed. Trunk extensors and flexors muscle strength was measured by an isokinetic dynamometer (Biodex System 4) during maximal concentric movement at 60°/s angular velocity [3,4]. Trunk extension (PT_ext_) and flexion peak torque (PT_flex_), expressed in Newton meters (Nm), were analyzed as absolute values as well as relative to bodyweight (Nm·kg^−1^). Patients with grade III obesity were found to have the lowest absolute trunk muscle strength compared to grade I patients, which were the ones displaying the highest muscle strength, both for extension (median [IQR]; grade I = 412.6 [85.1] vs. grade III = 230.3 [87.1]; *p* < 0.001) and flexion (grade I = 214.3 [84.4] vs. grade III = 103.1 [62.3]; *p* < 0.001). Compared with mildly obese patients, those with severe obesity also displayed lower relative muscle strength for both trunk extension (grade I = 5.0 [1.1] vs. grade III = 2.1 [0.8]; *p* < 0.001) and flexion (grade I = 2.5 [0.8] vs. grade III = 0.9 [0.6]; *p* < 0.001). Absolute trunk extension and flexion strength were also found to be inversely associated with body mass (PT_ext_: r = −0.16, *p* = 0.046; PT_flex_: r = −0.18, *p* = 0.025) and particularly with BMI (PT_ext_: r = −0.36, *p* < 0.001; PT_flex_: r = −0.44, *p* < 0.001) and %fat mass (PT_ext_: r = −0.56, *p* < 0.001; PT_flex_: r = −0.61, *p* < 0.001). Relative trunk muscle strength was also strongly and inversely associated with body mass (PT_ext_: r = −0.64, *p* < 0.001; PT_flex_: r = −0.62, *p* < 0.001), BMI (PT_ext_: r = −0.76, *p* < 0.001; PT_flex_: r = −0.79, *p* < 0.001), and %fat mass (PT_ext_: r = −0.81, *p* < 0.001; PT_flex_: r = −0.83, *p* < 0.001). As body mass increases, but mostly as excess adiposity increases, there is a substantial decrease in both absolute and relative trunk extension and flexion muscle strength. The magnitude of this inverse association is exacerbated when trunk muscle strength is normalized to bodyweight expressing functional strength.

**Funding:** This study was funded by the Fundação para a Ciência e a Tecnologia (FCT) (grant PTDC/DTP-DES/0968/2014) and the European Regional Development Fund (ERDF) through the Operational Competitiveness Programme (COMPETE) (grant POCI-01-0145-FEDER-016707). The study was developed in the Research Centre in Physical Activity, Health and Leisure (CIAFEL) funded by ERDF through COMPETE and by FCT (grant UIDB/00617/2020), and the Laboratory for Integrative and Translational Research in Population Health (ITR). Florêncio Diniz-Sousa, Lucas Veras, and Giorjines Boppre are supported by the FCT grants SFRH/BD/117622/2016, UI/BD/150673/2020, and SFRH/BD/146976/2019, respectively.

**References**

1. Pinto Pereira, S.M.; De Stavola, B.L.; Rogers, N.T.; Hardy, R.; Cooper, R.; Power, C. Adult obesity and mid-life physical functioning in two British birth cohorts: Investigating the mediating role of physical inactivity. *Int. J. Epidemiol.* **2020**, *49*, 845–856, https://doi.org/10.1093/ije/dyaa014.
2. Rogers, N.T.; Power, C.; Pinto Pereira S.M. Birthweight, lifetime obesity and physical functioning in mid-adulthood: A nationwide birth cohort study. *Int. J. Epidemiol.* **2020**, *49*, 657–665, https://doi.org/10.1093/ije/dyz120.
3. Estrázulas, J.A.; Estrázulas, J.A.; de Jesus, K.; de Jesus, K.; da Silva, R.A.; Dos Santos J.O.L. Evaluation isometric and isokinetic of trunk flexor and extensor muscles with isokinetic dynamometer: A systematic review. *Phys. Ther. Sport* **2020**, *45*, 93–102. https://doi.org/10.1016/j.ptsp.2020.06.008.
4. Müller, S.; Stoll, J.; Müller, J.; Mayer, F. Validity of isokinetic trunk measurements with respect to healthy adults, athletes and low back pain patients. *Isokinet. Exerc. Sci.* **2012**, *20*, 255–266, https://doi.org/10.3233/IES-2012-00482.

### 2.31. Physiological Characterization of a Typical CrossFit^®^ Benchmark in Experienced Crossfitters

Manoel Rios ^1,2,*^, Ana Sofia Monteiro ^1,2^, Klaus Magno Becker ^1,2^, Victor Machado-Reis ^3^, João Paulo Vilas-Boas ^1,2^, Daniel Moreira-Gonçalves ^4,5^ and Ricardo J. Fernandes ^1,2^

^1^ Center of Research, Education, Innovation and Intervention in Sport (CIFI2D), Faculty of Sport, University of Porto, Porto, Portugal; manoel.rios@hotmail.com (M.R); a.sofia.monteiro@gmail.com (A.S.M.); klausmagnobecker@gmail.com (K.M.B.); jpvb@fade.up.pt (J.P.V.B.); ricfer@fade.up.pt (R.J.F.)

^2^ Porto Biomechanics Laboratory, Faculty of Sport, University of Porto (LABIOMEP), Porto, Portugal

^3^ Research Center of Sports Sciences, Health Sciences & Human Development (CIDESD), University of Trás-os-Montes e Alto Douro (UTAD), Vila Real, Portugal; victormachadoreis@gmail.com

^4^ Research Center in Physical Activity, Health and Leisure (CIAFEL), Faculty of Sport, University of Porto (FADEUP), Portugal; danielmgon@fade.up.pt

^5^ Laboratory for Integrative and Translational Research in Population Health (ITR), Porto, Portugal

* Correspondence: manoel.rios@hotmail.com

One of the most popular CrossFit^®^ benchmarks is the Fran, a time-scored workout consisting of three rounds of 21–15–9 thrusters plus pull-up repetitions [1]. However, there is a lack of information concerning the involved physiological exertion, leading to difficulties in crossfitters performance optimization [2]. The aim of this study was to physiologically characterize the Fran workout. Ten highly trained crossfitters (six males and four females with 28.2 ± 6.2 vs. 25.5 ± 5.3 years, 82.3 ± 9.9 vs. 62.3 ± 2.4 kg of body mass, 175.8 ± 4.9 vs. 160.5 ± 3.9 cm of height and 26.5 ± 2.0 vs. 24.2 ± 0.8 of BMI, respectively) volunteered to participate. The breath-by-breath oxygen uptake and heart rate were continuously measured during the workout, capillary blood samples for lactate and glucose concentrations were collected from the earlobe before and during the 30 s transitions between rounds, and a 6–20 Borg scale was used to rate the perceived exertion during the workout. A paired-samples *t*-test was used to compare the assessed variables between baseline and in-between rounds (*p* < 0.05). The total workout duration and performance decreased from rounds 1 to 3 (77.3 ± 5.3 vs. 59.5 ± 7.4 vs. 40.2 ± 5.9 s and 0.55 ± 0.04 vs. 0.51 ± 0.06 vs. 0.46 ± 0.06 repetitions/s, respectively). During the Fran workout, the mean oxygen uptake presented similar results (34.3 ± 6.2 vs. 30 ± 5.3 vs. 22 ± 3.6 mL∙kg^−1^∙min^−1^), while the peak oxygen uptake decreased in round 2 and stabilized in round 3 (45.9 ± 4.5 vs. 43.9 ± 3.9 vs. 42.4 ± 3.7 mL∙kg^−1^∙min^−1^). Mean and peak heart rate were similar during the workout (158 ± 8 vs. 162 ± 10 vs. 162 ± 9 and 177 ± 9 vs. 177 ± 8 vs. 178 ± 6 b∙min^−1^), while the rate of perceived exertion was higher in rounds 2 and 3 (13 ± 2 vs. 15 ± 1 vs. 15.8 ± 2). Blood lactate concentrations increased during the workout (5.3 ± 1.4 vs. 9.1 ± 2.4 vs. 12.0 ± 2.4 mmol∙L^−1^), whilst blood glucose only increased in round 3 (102.4 ± 9.0 vs. 108.7 ± 14.5 vs. 114 ± 12.2 mg∙dL^−1^). The aerobic and anaerobic alactic energy system contributions decreased during the workout (51.7 ± 10.0 vs. 45.5 ± 7.8 vs. 31.5 ± 5.5 kJ and 29.2 ± 5.2 vs. 27.8 ± 5.1 vs. 24.2 ± 4.8 kJ, respectively) and the anaerobic lactic system kept its contribution stable (16.8 ± 5.3 vs. 16.9 ± 10.4 vs. 12.1 ± 4.4 kJ), with a decrease being observed in energy expenditure in round 3 (97.7 ± 17 vs. 90.1 ± 18.7 vs. 67.8 ± 12.0 kJ). Regarding metabolic power, an increase was observed along the workout (1.3 ± 0.3 vs. 1.6 ± 0.3 vs. 1.8 ± 0.4 kJ). The current data evidenced that the Fran workout is a high-intensity effort, associated with elevated metabolic demand. In addition, since it is characterized by predominantly anaerobic energy use, crossfitters’ training should be based on rigorous monitoring of specific workouts and adjusted balancing of consecutive workloads on anaerobic and aerobic exertions.

**Funding:** This work was supported by the Portuguese Foundation for Science and Technology (FCT) to MR PhD scholarship (2021.04701.BD).

**References**

1. Tibana, R.A.; De Sousa, N.M.F.; Cunha, G.V.; Prestes, J.; Fett, C.; Gabbett, T.J.; Voltarelli, F.A. Validity of session rating perceived exertion method for quantifying internal training load during high-intensity functional training. *Sports* **2018**, *6*, 68.
2. Leitão, L.; Dias, M.; Campos, Y.; Vieira, J.G.; Sant’Ana, L.; Telles, L.G.; Tavares, C; Mazini, M.; Novaes, F.; Vianna, J. Physical and Physiological Predictors of FRAN CrossFit^®^ WOD Athlete’s Performance. *Int. J. Environ. Res. Public Health* **2021**, *18*, 4070.

### 2.32. Centrality of Dynamic and Kinematic Whole-Body Variables in Elite and Non-Elite Subjects in Standard Maximum Vertical Jump

**Carlos Rodrigues ^1,2^, Miguel Correia ^1,2^, João Abrantes ^3^, Marco Rodrigues ^4^ and Jurandir Nadal ^5^**

^1^ INESC TEC—Technology & Science Associate Laboratory; carlos.b.rodrigues@inesctec.pt

^2^ FEUP—Faculty of Engineering of the University of Porto; mcorreia@fe.up.pt

^3^ MovLab—Interactions and Interfaces Lab; joao.mcs.abrantes@ulusofona.pt

^4^ DES—Department of Electronic and Systems, Federal University of Pernambuco; benedetti@ufpe.br

^5^ PEB—Biomedical Engineering Program, Federal University of Rio de Janeiro; jn@peb.ufrj.br

Countermovement (CM) is a natural human action with eccentric movement preceding concentric movement for efficient submaximal and powerful maximal execution [1]. Lower-limb CM can be observed in gait, running, and jumping, with higher expression and accessibility in the standard maximum vertical jump (MVJ) with long CM in the countermovement jump (CMJ) and short CM at drop jump (DJ) in comparison with the squat jump (SJ) without CM [2], and this standard MVJ is recurrently used in track and field, volleyball, basketball, handball, football, tennis, and many other sports [3], with an open issue on CM strength and conditioning. High dimensionality, time dependence, and the strong correlation of human movement dynamic and kinematic variables point to the need for the dimensional reduction and detection of central variables with dominant correlation [4]. Moreover, if the objective is to determine how an athlete can improve performance, it is essential to detect central physical quantities to be trained [5]. For this purpose, we have applied graph algorithms for the detection of central variables according to the higher node degree centrality of bivariate correlations between dynamic and kinematic whole-body variables in elite and non-elite subjects during standard MVJ. Fifty-one dynamic and kinematic variables were selected for analysis, grouped into three communities, namely (i) time-force-impulse (t-F-I), (ii) force-velocity-power (F-v-P), and (iii) force-displacement-work (F-z-W) of the whole-body center of gravity during the best MVJ type (SJ, CMJ, DJ) of three trial repetitions for each elite (E) male group subject (nE = 16) of the Portuguese national volleyball team and non-elite (NE) male group subject (nNE = 6) of sports degree students without previous injuries, specific training, or sport modality. Dynamic and kinematic variables were subjected to bivariate correlation for each group (E, NE), MVJ (SJ, CMJ, DJ), and community (i, ii, iii), with statistical significance assessments at the 5% significance level. Statistically significant correlations were processed using graph algorithms for degree centrality and graph density assessment. Global subgraph densities presented a similar profile on E and NE groups for each MVJ type, which can be associated with the same variable structure for each MVJ type on the E and NE groups assessed. Nevertheless, when analyzed separately, each subgraph presents clear differences in the E and NE groups concerning their density at SJ, CMJ, and DJ. Thus, main differences were detected on t, t-I, and F-I graph densities, whereas F, t-F, and I graphs presented similar behavior at E and NE in SJ, CMJ, and DJ comparisons. Concerning v, P, v-F, F-P, v-P, and F-v-P graphs, they all presented different densities at E and NE for comparisons on SJ, CMJ, and DJ. Finally, despite z, W, F-z, z-W, and F-z-W presenting different graph densities, upon comparison, SJ, CMJ, DJ on E and NE, F-W present similar graph densities. Different degree centrality pointed to variables with training interest.

**Funding:** This research was funded by EACEA.

**References**

1. Komi, P.V.; Ishikawa, M.; Linnamo, V. Identification of stretch-shortening cycles in different sports. *Port. J. Sport Sci.* **2011**, *11*, 31–34.
2. Asmussen, E. Storage of Elastic Energy in Skeletal Muscles in Man. *Acta Physiol. Scand*. **1974**, *91*, 385–392.
3. Knudson, D. *Fundamentals of Biomechanics*, 2nd ed.; Springer: New York, NY, USA, 2008; pp. 23–165.
4. Chau, T. A review of analytical techniques for gait data. Part 1: Fuzzy, statistical and fractal methods. *Gait Posture* **2001**, *13*, 49–66.
5. Zatsiorsky, V.M.; Kraemer, W.J. *Science and Practice of Strength Training*, 2nd ed.; Human Kinetics: Champaign, IL, USA, 2006; pp. 17–46.

### 2.33. Preferential Whole-Body Dynamic and Kinematic Variable Coupling in Elite and Non-Elite Subjects in Standard Maximum Vertical Jump

**Carlos Rodrigues ^1,2^, Miguel Correia ^1,2^, João Abrantes ^3^, Marco Rodrigues ^4^ and Jurandir Nadal ^5^**

^1^ INESC TEC—Technology & Science Associate Laboratory; carlos.b.rodrigues@inesctec.pt

^2^ FEUP—Faculty of Engineering of the University of Porto; mcorreia@fe.up.pt

^3^ MovLab—Interactions and Interfaces Lab; joao.mcs.abrantes@ulusofona.pt

^4^ DES—Department of Electronic and Systems, Federal University of Pernambuco; benedetti@ufpe.br

^5^ PEB—Biomedical Engineering Program, Federal University of Rio de Janeiro; jn@peb.ufrj.br

Countermovement (CM) has been used in strength and conditioning, sports, and physical assessment [1,2] as a natural human action, with plyometrics preceding muscle tendon unit shortening, towards more efficient submaximal and powerful maximal activity [3], with an open question on biomechanical factors determining CM performance across tasks, subjects’ abilities, and training. According to higher expression and accessibility, CM has been assessed in lower limbs in the standard maximum vertical jump (MVJ) with long CM in the countermovement jump (CMJ) and short CM in the drop jump (DJ) in comparison with the squat jump (SJ) with no CM [4]. Due to high dimensionality, time dependence, and strong correlations between human movement dynamics and kinematic variables, preferential attachment presents as a promising graph technique to detect the most frequent correlations of CM with each MVJ type as natural phenomena in relation to trained/untrained and subjects’ abilities. Thus, we have applied pathfinding graph algorithms for the detection of variable couplings corresponding to the most frequent bivariate correlations between dynamic and kinematic whole-body variables in elite and non-elite subjects during standard MVJ. Eighteen variables from (i) time-force-impulse (t-F-I), twenty-four from (ii) force-velocity-power (F-v-P), and twenty from (iii) force-displacement-work (F-z-W) of the whole-body center of gravity were analyzed using graph communities of bivariate correlations of the best execution of three repetitions from SJ, CMJ, and DJ for each elite (E) male group subject (nE = 16) of the Portuguese national volleyball team and non-elite (NE) male group subject (nNE = 6) of sports degree students without previous injuries, specific training, or sport modality. Dynamic and kinematic variables were subjected to bivariate correlation for each group (E, NE), MVJ (SJ, CMJ, DJ), and community for a total of 152 (i), 248 (ii), and 162 (iii) correlations, with statistical significance assessment at the 5% significance level. Preferential attachment was computed for each graph community with pathfinding algorithms for the detection of the most frequent correlations of each MVJ type for NE and E. Preferential attachment analysis presented clear distinctions, particularly for t-F, t-I, F-I, and z-F with lower statistically significant variable coupling (*p* < 0.05) in NE in relation to E group. Several links corresponding to coupling correlations were simultaneously detected in NE and E groups on specific edges, corresponding to shared relations between E and NE groups. On the other hand, a larger number of coupling correlations were detected exclusively in the NE group, with a reduced number of coupling correlations exclusively in E. Together, these results point to the existence of common relations in both NE and E groups, with NE presenting a larger number of non-specific relations to the E group, while the E group presents a small number of specific relations not shared with the NE group, and this a potential line for CM strength and conditioning.

**Funding:** This research was funded by EACEA.

**References**

1. Zatsiorsky, V.M.; Kraemer, W.J. *Science and Practice of Strength Training*, 2nd ed.; Human Kinetics: Champaign, IL, USA, 2006; pp. 17–46.
2. Knudson, D. *Fundamentals of Biomechanics*, 2nd ed.; Springer: New York, NY, USA, 2008; pp. 23–165.
3. Komi, P.V.; Ishikawa, M.; Linnamo, V. Identification of stretch-shortening cycles in different sports. *Port. J. Sport Sci.* **2011**, *11*, 31–34.
4. Asmussen, E. Storage of Elastic Energy in Skeletal Muscles in Man. *Acta Physiol. Scand*. **1974**, *91*, 385–392.

### 2.34. Center of Pressure Velocity Analysis: Comparison between Standing Posture and Cognitive Dual-Task in Young Adults

Marina Saraiva ^1,2,^*, João Paulo Vilas-Boas ^2,3^ and Maria António Castro ^1,4,5^

^1^ RoboCorp Laboratory, i2A, Polytechnic Institute of Coimbra, 3046-854 Coimbra, Portugal; marina.saraiva@outlook.com

^2^ Faculty of Sports, University of Porto, 4200-450 Porto, Portugal

^3^ LABIOMEP-UP, Faculty of Sports and CIFI2D, the University of Porto, 4200-450 Porto, Portugal; jpvb@fade.up.pt

^4^ Department of Mechanical Engineering, University of Coimbra, CEMMPRE, 3030-788 Coimbra, Portugal

^5^ Sector of Physiotherapy, School of Health Sciences, Polytechnic Institute of Leiria, 2411-901 Leiria, Portugal; maria.castro@ipleiria.pt

* Correspondence: marina.saraiva@outlook.com

Center of pressure (CoP) analysis is often used to assess postural control and understand motor control mechanisms while simultaneously performing different tasks [1]. The CoP velocity can be considered an indicator of the efficiency of postural control, and it is a sensitive measure to detect changes in postural control [2,3]. The purpose of this study was to evaluate the center of pressure velocity during quiet standing posture and a cognitive dual-task in young adults. Thirty-six young adults (23.08 ± 3.92 years) participated in this study. Each subject was requested to perform two tasks: Standing quietly (single task—ST) and keeping a quiet standing posture while playing a mental game based on arithmetic or memory tasks on their smartphone (cognitive dual-task—cog-DT) on a force plate for 60 s. The mean total velocity of CoP (MVELO_CoP) and the mean anterior–posterior and medial–lateral velocities of CoP (MVELO_CoP-AP and MVELO_CoP-ML, respectively) were acquired through the Bertec^®^ force platform, and the data were assessed with a Matlab routine. Statistical analyses were performed using IBM-SPSS (version 25.0), and the level of significance was set at *p* < 0.05. To compare CoP velocity between single and cognitive dual-task, we used the related-sample Wilcoxon signed-rank test. The data were presented as the median and interquartile range (IQR). The MVELO_CoP (Cog-DT: 509.41 (453.83–603.70) mm/s vs. ST: 481.40 (439.66–561.25) mm/s; *p* < 0.000), MVELO_CoP-AP (cog-DT: 389.62 (349.89–454.85) mm/s vs. ST: 368.85 (330.20–431.38) mm/s; *p* < 0.000), and MVELO_CoP-ML (cog-DT: 252.53 (224.13–305.83) mm/s vs. ST: 243.53 (215.31–280.97) mm/s; *p* < 0.000) increased during the cognitive dual-task compared to the single task. The results suggest that maintaining a quiet posture while playing a mental game on a smartphone decreases young adults’ ability to control upright posture compared to maintaining quiet standing posture only. Furthermore, these apparent changes in a young sample may predict more significant changes in postural control in old age, namely, more risk of falls.

**Funding:** This research was funded by Fundação para a Ciência e a Tecnologia, Portugal, grant number 2021.08571.BD.

**References**

1. Chen, B.; Liu, P.; Xiao, F.; Liu, Z.; Wang, Y. Review of the Upright Balance Assessment Based on the Force Plate. *Int. J. Environ. Res. Public Health* **2021**, *18*, 2696.
2. Palmieri, R.M.; Ingersoll, C.D.; Stone, M.B.; Krause, B.A.; Center of pressure parameters used in the assessment of postural control. *J. Sport Rehabil.* **2002**, *11*, 51–66.
3. Roma, E.; Gobbo, S.; Bullo, V.; Spolaor, F.; Sawacha, Z.; Duregon, F.; Bianchini, G.; Doria, E.; Alberton, C.L.; Bocalini, D.S.; et al. Influence of age on postural control during dual task: A center of pressure motion and electromyographic analysis. *Aging Clin. Exp. Res.* **2021**, *34*, 137–149.

### 2.35. Differences in Worst-Case Scenarios Using Rolling Average between Official and Non-Official Matches in Elite Futsal

**Konstantinos Spyrou ^1,2,4^, Tomás T. Freitas ^1,2,3,4^, Elena Marín-Cascales ^4^, Rubén Herrero-Carrasco ^5^ and Pedro E. Alcaraz ^1,4^**

^1^ UCAM Research Center for High Performance Sport, Catholic University of Murcia, Murcia, Spain; kspyrou@ucam.edu

^2^ Faculty of Sport Sciences, Catholic University of Murcia, Murcia, Spain

^3^ NAR—Nucleus of High Performance in Sport, São Paulo, Brazil

^4^ Strength and Conditioning Society, Rome, Italy

^5^ Research Group Murcia Soccer Federation, Murcia, Spain

This study aimed to compare the Worst-Case Scenarios (WCS) between official (OFF) and non-official (Non-OFF) matches and among different time-periods in an elite futsal team [1,2]. Twenty-six games were classified as either OFF (n = 13) or Non-OFF (n = 13). The WCS were calculated based on player load per minute (PL·min^−1^) using rolling averages (ROLL) and four-length epochs (30-s, 1-, 3-, and 5-min). Significant and small differences were found in PL·min^−1^ in 30-s (*p* = 0.001; ES = −0.53) and 1-min (*p* = 0.001; ES = −0.47) between matches, but non-significant and small to trivial differences were observed in 3-min (*p* = 0.060; ES = −0.23) and 5-min (*p* = 0.605; ES = −0.06). Significant differences in PL·min^−1^ were observed with increasing time-windows (*p* = 0.001). In summary, OFF matches present higher WCS than Non-OFF when considering short time periods. From an applied perspective, based on the present data, sport practitioners are advised to use short time-periods (i.e., 30-s and 1-min) to quantify WCS in futsal as these were the only found to be able to discriminate between different types of matches (i.e., OFF and Non-OFF). Conversely, larger time-periods (i.e., 3- and 5-min) appear to obscure the “actual intensity” that the players are exposed to.

**Funding:** This research received no external funding.

**References**

1. Novak, A.R.; Impellizzeri, F.M.; Trivedi, A.; Coutts, A.J.; McCall, A. Analysis of the worst-case scenarios in an elite football team: Towards a better understanding and application. *J. Sports Sci.* **2021**, *39*, 1850–1859. https://doi.org/10.1080/02640414.2021.1902138.
2. Spyrou, K.; Freitas, T.T.; Marín-Cascales, E.; Herrero-Carrasco, R.; Alcaraz, P.E. External match load and the influence of contextual factors in elite futsal. *Biol. Sport* **2021**, *39*, 349–354. https://doi.org/10.5114/biolsport.2022.105332.

### 2.36. Strength Training Practices in Elite Futsal: A Survey of Current Practitioners

**Konstantinos Spyrou ^1,2,4^, Tomás T. Freitas ^1,2,3,4^, Elena Marín-Cascales ^4^, Rubén Herrero-Carrasco ^5^ and Pedro E. Alcaraz ^1,4^**

^1^ UCAM Research Center for High Performance Sport, Catholic University of Murcia, Murcia, Spain; kspyrou@ucam.edu

^2^ Faculty of Sport Sciences, Catholic University of Murcia, Murcia, Spain

^3^ NAR—Nucleus of High Performance in Sport, São Paulo, Brazil

^4^ Strength and Conditioning Society, Rome, Italy

^5^ Research Group Murcia Soccer Federation, Murcia, Spain

Strength training (ST) is a necessary component to enhance physical performance and reduce injuries in sports [1,2]. To the authors’ knowledge, this the first study investigating the current practices in ST in futsal. Thirty-seven strength and conditioning coaches (S&Cc) currently working in the first, second, or 2n dB divisions from two of the world’s leading countries in futsal (Spain, n = 24, and Portugal, n = 13), completed an online questionnaire consisting of two parts: Background information and ST characteristics. All questions (n = 12) were closed, providing respondents with a predetermined set of answers with a comment box “other”. Most questions allowed more than one response because coaches could report using multiple methods. Responses were analyzed using frequency analysis for each question and were presented as absolute frequencies and percentages. Mean ± standard deviation was calculated for a single question: “The importance of strength in futsal” as a 1–5 Likert scale (1 = not very important, 5 = extremely important) was used. From the total sample, 76.6% of the coaches work in the men’s first Division, 5.4% in the second Division, 10.8% in the 2n dB Division, and 16.2% in the women’s first Division. The age of the practitioners and the working experience ranged from 20 to 50+ years old and from 1 to 8+ years, respectively. Overall, ST in futsal was considered as “extremely important” (4.8 ± 0.4). During normal weeks, the majority of the S&Cc (43.2%) reported completing the first ST on the morning of Match Day (MD) + 2, followed by the afternoon of MD+1 (16.2%), the morning of MD+3 (16.2%), and the afternoon of MD+2 (13.5%). Most ST sessions were reported to last from 16 to 30 min (45.9%), followed by 31–45 min (29.7%), 46–60 min (16.2%), 0–15 min (2.7%), 61–75 min (2.7%), and >76 min (2.7%), and focused on full-body training (i.e., upper and lower limbs) (73%) and core (67.6%) exercises. During congested periods, 18.9% of the S&Cc reported not using ST. Amongst those who do, most indicated that ST is performed in the morning of MD+2 (27.9%), afternoon of MD+1 (16.2%) or MD+2 (16.2%), morning of MD+1 (10.8%), and, lastly, morning or afternoon of MD+3 (2.7%). S&Cc reported no differences of the sessions’ duration and exercise training in congested periods when compared to normal weeks. Interestingly, ST is prescribed according to %1RM–XRM (59.5%), velocity-based training (21.7%), repetitions in reserve (18.9%), until failure (10.8%), and circuit training (2.7%). The main aspects to improve related to ST, as reported by the S&Cc, were “Better Monitoring” (73.5%), “More Individualized” (62.2%), “Better Facilities” (55.6%), “More Staff” (35.1%), and “More Time” (10.8%). In conclusion, the current practices of S&Cc suggest that ST plays a crucial role in physical preparation in futsal, and is mostly performed on MD+2, with sessions combining upper and lower body and core exercises that last 16–30 min. 

**Funding:** This research received no external funding.

**References**

1. Case, M.J.; Knudson, D.V.; Downey, D.L. Barbell squat relative strength as an identifier for lower extremity injury in collegiate athletes. *J. Strength Cond. Res.* **2020**, *34*, 1249–1253. https://doi.org/10.1519/JSC.0000000000003554.
2. Spyrou, K.; Freitas, T.T.; Marín-Cascales, E.; Alcaraz, P.E. Physical and physiological match-play demands and player characteristics in futsal: A systematic review. *Front. Psychol.* 2020, *11*, 2870. https://doi.org/10.3389/fpsyg.2020.569897.

### 2.37. Comparison of Neuromuscular Fatigue between Strength and Power Training Modalities within the Microcycle

**Sajith M.A.S. Udayanga, Antonio Martínez-Serrano, Pedro E. Alcaraz and Linda Chung**

Research Center for High Performance Sport, Universidad Católica de Murcia, Spain; sumarasingha@alu.ucam.edu

Fatigue is commonly defined as an exercise-induced reduction in the ability to exert muscle force or power [1]. Fatigue is a complex and multifaceted phenomenon that has a variety of mechanisms that affect the central nervous system (central fatigue) and skeletal muscle (peripheral fatigue) [1,2]. Therefore, it is important to monitor and understand the behavior of the neuromuscular fatigue during training cycles to avoid the negative outcomes of non-functional overreaching and overtraining. Hence, this study aimed to compare neuromuscular fatigue markers during strength (ST) and power (PT) training sessions, which occurred 48 h after a fatigue-induced (modified beast protocol, MBP) session within the micro training cycle. Eleven physically active participants underwent one-repetition maximum (1 RM) and an optimal load (OL) test during the familiarization session. After a 7-day resting period, participants performed 45 min of MBP. Forty-eight hours (H) later, they completed the ST session, consisting of half squat, bench press, and hip thrust exercises (ST: four 5-reps, 90% 1 RM, 4 min rest between sets; PT: four 5-reps, OL, 3 min rest between sets). Countermovement jump height (CMJ) and maximum voluntary isometric contraction peak force (MVC) tests were performed before and after MBP and ST session, post-6H and -24H. After a 7-day washout period, participants completed the same training protocol but with the PT session. A one-way repeated-measure ANOVA test was conducted to determine significant differences between time points in each trial. There was a significant decrease in CMJ at MBP (*p* = 0.026, ES = −0.31), Pre-RT (*p* = 0.039, ES = −0.20) and Post-RT (*p* = 0.045, ES = −0.21) in PT, and Pre-RT (*p* = 0.021, ES = −0.30) and Post-RT (*p* = 0.020, ES = −0.46) in ST modality compared to the respective baseline values. Compared to baseline values, there was a significant reduction in MVC at MBP (ST: *p* = 0.038, ES = −0.33; PT: *p* = 0.014, ES = −0.38) in both training modalities. There was a tendency towards a significant decrease in MVC at Post-RT (*p* = 0.063, ES = −0.45) in PT. Interestingly, the ST trial (*p* = 0.020, ES = −0.43) showed a significant decrease in MVC at Post−24H, but not for the PT trial (*p* = 0.087, ES = −0.26) compared to baseline values. In conclusion, the present study showed that performing a PT session under fatigued conditions may generate less neuromuscular fatigue and better recovery compared to the ST session within the micro training cycle.

**Funding:** This research received no external funding.

**References**

1. Gandevia, S.C. Spinal and supraspinal factors in human muscle fatigue. *Physiol. Rev.* **2001**, *81*, 1725–1789.
2. Halson, S.L. Monitoring training load to understand fatigue in athletes. *Sports Med.* **2014**, *44*, 139–147.

### 2.38. Comparison of Poincaré Plot (HRV) Parameter’s Recovery between Strength and Power Training under Fatigued Conditions within the Microcycle

**Sajith M.A.S. Udayanga, Antonio Martínez-Serrano, Pedro E. Alcaraz and Linda Chung**

Research Center for High Performance Sport, Universidad Católica de Murcia, Spain; sumarasingha@alu.ucam.edu

The Poincaré plot is a non-linear method to evaluate Heart Rate Variability (HRV), where standard deviation (SD) transverse axis (SD1—proportional to parasympathetic activity), longitudinal axis (SD2—inversely proportional to sympathetic activity), and SD2/SD1 ratio parameters are widely used. The newly introduced stress score index (SS) parameter also facilitates (SS = 1000 × 1/SD2) the understanding of changes in cardiac sympathetic activity [1,2,3]. This study aimed to compare the recovery time course of SD1, SD2, SD2/SD1, and SS parameters within the microcycle, between strength (ST) and power training (PT) trials under a fatigued condition. Eleven physically active subjects participated. In the familiarization session, subjects performed one-repetition maximum (1 RM) and optimal load (OL) tests. After 7 days, subjects completed a 45-min modified beast protocol (MBP). Forty-eight hours (H) later, they performed the ST modality, which consisted of half squat, bench press, and hip thrust exercises (ST: Four 5-reps, 90% 1 RM, 4 min rest between sets; PT: Four 5-reps, OL, 3 min rest between sets). HRV data were recorded for 10 min in a quiet room with the subject in the supine position, before and after the MBP and RT session, post-6H, -24H, and -48H. The last 5 min of HRV data were analyzed. After a one-week washout period, subjects returned to perform the same protocol but with the PT modality. A one-way repeated measure ANOVA test was used to determine the significant difference between time points in each trial. Compared to pre-values, a significant decrease in SD1 was detected at MBP (*p* < 0.001), Pre-RT (*p* = 0.002), Post-RT (*p* = 0.005), Post-6H (*p* < 0.001), and Post-24H (*p* = 0.001) in the ST trial and MBP (*p* < 0.001), Pre-RT (*p* = 0.001), Post-RT (*p* < 0.001), and Post-6H (*p* = 0.040) in the PT trial. A significant decrease in SD2 was observed at Pre-RT (*p* = 0.012) and Post-6H (*p* = 0.013) in the ST trial and MBP (*p* = 0.020) in the PT trial compared to pre-values. Interestingly, SD2/SD1 ratio parameter results did not show significant pre-post differences between time points in both trials. However, the effect size (ES) data showed that the SD2/SD1 ratio recovered at Post-24H (ES = −0.40) in PT, whereas it did not recover at Post-48H (ES = 0.59) in ST. Compared to pre-values, there was a significant increase in SS after MBP (ST: *p* = 0.008, PT: *p* = 0.010) and post-RT (ST: *p* = 0.010, PT: *p* = 0.034) in both modalities. Furthermore, significant changes were detected in pre-RT (*p* = 0.012) and post-6H (*p* = 0.001) in the ST trial. Interestingly, ES results showed that SS of both training modalities did not recover at post-48H. Even though not fully recovered, PT showed a better recovery level (ES = 0.14) than ST (ES = 0.25) at post-48H. In conclusion, our data revealed that performing a PT session under fatigued conditions may help facilitate the recovery process compared to the ST session within the micro training cycle.

**Funding:** This research received no external funding.

**References**

1. Orellana, J.N.; de la Cruz Torres, B.; Cachadiña, E.S.; de Hoyo, M.; Cobo, S.D. Two new indexes for the assessment of autonomic balance in elite soccer players. *Int. J. Sports Physiol. Perform.* **2015**, *10*, 452–457.
2. Navarro-Lomas, G.; Jurado-Fasoli, L.; Castillo, M.J.; Femia, P.; Amaro-Gahete, F.J. Assessment of autonomous nerve system through non-linear heart rate variability outcomes in sedentary healthy adults. *PeerJ* **2020**, *8*, e10178.
3. Henriques, T.; Ribeiro, M.; Teixeira, A.; Castro, L.; Antunes, L.; Costa-Santos, C. Nonlinear methods most applied to heart-rate time series: A review. *Entropy* **2020**, *22*, 309.

### 2.39. Are the Effects of a Multicomponent Exercise Training on Knee Muscle Strength after Bariatric Surgery Mediated by Lean Mass?

Lucas Veras ^1,2,^*, Florêncio Diniz-Sousa1 ^1,2^, Giorjines Boppre ^1,2^, Vítor Devezas ^3^, Hugo Santos-Sousa ^3^, John Preto ^3^, João Paulo Vilas-Boas ^4,5^, Leandro Machado ^4,5^, José Oliveira ^1,2^ and Hélder Fonseca ^1,2^

^1^ Research Centre in Physical Activity, Health and Leisure (CIAFEL), Faculty of Sport, University of Porto, Porto, Portugal

^2^ Laboratory for Integrative and Translational Research in Population Health (ITR), University of Porto, Porto, Portugal

^3^ Centro de Responsabilidade Integrado de Obesidade (CRIO), Centro Hospitalar Universitário de São João, Porto, Portugal

^4^ Centre of Research, Education, Innovation and Intervention in Sport (CIFI2D), Faculty of Sport, University of Porto, Porto, Portugal

^5^ Porto Biomechanics Laboratory (LABIOMEP), University of Porto, Porto, Portugal

* lucasdsveras@gmail.com

Bariatric surgery (BS) is an effective treatment for severe obesity and associated comorbidities [1]. Nevertheless, BS is also associated with lean mass losses and muscle strength reduction [2,3]. Exercise training programs have been demonstrated to be a feasible and safe adjunct therapy for BS patients to help counteract these negative effects [4]. Therefore, we aimed to determine whether the multicomponent exercise program effects on knee muscle strength after BS are mediated by lean mass. This is an ancillary study of a randomized clinical trial registered at ClinicalTrials.gov (NCT02843048). Forty-six patients (9 males) aged 42.6 ± 9.3 years with a body mass index of 44.1 ± 4.6kg·m^−2^ were allocated to either a control (CG; BS + standard medical care; n = 15) or exercise group (EG; BS + standard medical care + multicomponent exercise program; n = 31) and were followed for 1 year after BS. The multicomponent exercise program started 1 month after surgery and consisted of 3 d/week, 75-min-duration multicomponent exercise sessions that included multidirectional jumps, balance, and resistance exercises. Dominant lower limb knee extensor and flexor muscle strength was measured by an isokinetic dynamometer (Biodex System 4 Pro) during maximal concentric movement at 60°/s angular velocity, between 0° and 90° of knee flexion. Knee extension and flexion peak torque were expressed as the percentage change from baseline to 1-year post-BS. Body composition was assessed through dual-energy X-ray absorptiometry (Hologic Explorer QDR). Whole-body lean mass and lower-limb lean mass were tested as mediators of the treatment effect on both knee extension and flexion. Total, direct, and indirect effects were estimated, with 95% confidence intervals (95%CI) obtained with 5000 bootstrap resamples. The effect size was expressed as the partially standardized indirect effect (ab_ps_). Compared to baseline, CG and EG muscle strength significantly decreased 1-year post-BS on both knee extension (CG: ∆ = −12.7%, *p* < 0.001; EG: ∆ = −14.8%, *p* < 0.001) and flexion (CG: ∆ = −7.2%, *p* < 0.001; EG: ∆ = −3.4%, *p* < 0.001). The treatment effect on the mediator was significant on whole-body lean mass (β = 3.322, 95%CI = 0.480 to 6.220, *p* = 0.026), while no significant effect was observed on lower-limb lean mass (β = 2.712, 95%CI = −0.901 to 6.487, *p* = 0.858). Whole-body lean mass had no significant indirect effect on knee extension (β = 0.068, 95%CI = −8.182 to 6.746, *p* = 0.985, ab_ps_ = 0.003) or knee flexion (β = −0.482, 95%CI = −7.848 to 4.552, *p* = 0.874, ab_ps_ = −0.022). One year after BS, both control and exercise patients showed a significant decrease in knee muscle strength. The multicomponent exercise program exerted a significant effect on whole-body lean mass, but that effect was not able to influence either knee extension or flexion muscle strength.

**Funding:** This study was funded by the Fundação para a Ciência e a Tecnologia (FCT) (grant PTDC/DTP-DES/0968/2014) and the European Regional Development Fund (ERDF) through the Operational Competitiveness Programme (COMPETE) (grant POCI-01-0145-FEDER-016707). The study was developed in the Research Centre in Physical Activity, Health and Leisure (CIAFEL) funded by ERDF through COMPETE and by FCT (grant UIDB/00617/2020), and the Laboratory for Integrative and Translational Research in Population Health (ITR). Lucas Veras, Florêncio Diniz-Sousa and Giorjines Boppre are supported by the FCT grants UI/BD/150673/2020, SFRH/BD/117622/2016, and SFRH/BD/146976/2019, respectively.

**References**

1. Jabbour, G.; Salman, A. Bariatric surgery in adults with obesity: The impact on performance, metabolism and health indices. *Obes. Surg*. **2021**, *31*, 1767–1789.
2. O’Brien, P.E.; Hindle, A.; Brennan, L.; Skinner, S.; Burton, P.; Smith, A.; Crosthwaite, G.; Brown, W. Long-term outcomes after bariatric surgery: A systematic review and meta-analysis of weight loss at 10 or more years for all bariatric procedures and a single-center review of 20-year outcomes after adjustable gastric banding. *Obes. Surg.* **2019**, *29*, 3–14.
3. Hue, O.; Berrigan, F.; Simoneau, M.; Marcotte, J.; Marceau, P.; Marceau, S.; Tremblay, A.; Teasdale, N. Muscle force and force control after weight loss in obese and morbidly obese men. *Obes. Surg*. **2008**, *18*, 1112–1118.
4. Boppre, G.; Diniz-Sousa, F.; Veras, L.; Oliveira, J.; Fonseca, H. Can exercise promote additional benefits on body composition in patients with obesity after bariatric surgery? A systematic review and meta-analysis of randomized controlled trials. *Obes. Sci. Pract*. **2021**, *8*, 112–123.

### 2.40. Use of Triaxial Accelerometry during Flamenco Footwork Technique

**N** **ingyi Z** **hang ^1^, Sebastián Gómez-Lozano ^1^ and Alfonso Vargas-Macías ^2^**

^1^ Performing Arts Research Group-Faculty of Sport. San Antonio Catholic University, Murcia, Spain; sglozano@ucam.edu

^2^ Telethusa Centre for Flamenco Research, Cádiz, Spain; vargas@flamencoinvestigacion.es

The high levels of effort required to perform the flamenco dance may cause chronic repetitive pain and injuries during the practice of footwork dancing. Therefore, the aim of this study was to quantify the external load of the flamenco footwork zap-3 via triaxial accelerometry in the form of PlayerLoad and utilize it to compare loading on the lumbar vertebra, cervical spine, and the lower limbs, as well as explore the effect on different positions and axes. Six professional flamenco dancers (age: 38.83 ± 7.96 years; height: 1.67 ± 0.10 m; weight: 63.33 ± 6.38; BMI: 22.52 ± 5.69 kg) completed 15-s Zap-3 footwork at a 240 bpm speed level. Tri-axial accelerometry was positioned at the ankle of the dominant foot (DA), the fifth lumbar vertebra (L5), and the seventh cervical spine (C7) and used to calculate the accumulated Total PlayerLoad (PLtotal), uni-axial Playerload (anteroposterior (PLap), mediolateral (PLml), and vertical (PLv)), and uni-axial contributions of the anterior-posterior (PLap%), medial-lateral (PLml%), and vertical (PLv%) planes. The accumulated PLtotal was calculated at C7, L5, and DA, defined as the square root of the sum of the squared instantaneous rate of change in acceleration in each of the three vectors (medial-lateral (PLml), anterior-posterior (PLap), and vertical (PLv)) and divided by 100. The percentage contribution of each uni-axial PlayerLoad vector (PLml%, PLap%, and PLv%) was also quantified by dividing the individual uniaxial PlayerLoad value by PLTotal and multiplying that value by 100. Regarding PLtotal, a significant main effect for the position (*p* < 0.001) was identified. Post-hoc analyses revealed that PLtotal was higher at DA (264.24 ± 32.42 au; CI: 245.97–282.51 au) compared with C7 (49.40 ± 11.69 au; CI = 31.14–67.67 au; *p* < 0.001; d = 8.82) and L5 (56.45 ± 11.59 au; CI = 38.18–74.72 au; *p* < 0.001; d = 8.55), respectively. There was no significant difference between L5 and C7 (*p* > 0.05). For the uni-axial contribution, PLml% represents a significantly larger contribution to PLtotal compared with PLv% and PLap% (*p* < 0.001). There was no significant main effect for position (*p* > 0.05) in any uni-axis. However, the position × uni-axial contribution interaction (*p* < 0.001) demonstrated that PLml% at C7 was significantly higher than both L5 and DA (*p* < 0.001); PLv% at DA was significantly higher than both C7 and L5 (*p* < 0.001); PLap% at L5 was significantly higher than both DA and C7 (*p* < 0.001). In conclusion, the authors deemed that, for professional flamenco dancers, the ankle bears the highest external load during zap-3 flamenco footwork, while the external load in the cervical spine and lumbar still exist because of the significant amount of vibration, and this may be one of the reasons behind pain or injury in professional flamenco dancers. However, it is necessary for further research to focus on the absorption of the impact of ground reaction forces in flamenco dancers.

**Funding:** This research received no external funding.

